# Activation of the unfolded protein response in high glucose treated endothelial cells is mediated by methylglyoxal

**DOI:** 10.1038/s41598-019-44358-1

**Published:** 2019-05-27

**Authors:** Zehra Irshad, Mingzhan Xue, Amal Ashour, James R. Larkin, Paul J. Thornalley, Naila Rabbani

**Affiliations:** 1 Clinical Sciences Research Laboratories, Warwick Medical School, University of Warwick, University Hospital, Coventry, CV2 2DX UK; 2 Speciality Clinics, University Dental Hospital, Taif Dental College, Taif University, Al-hawiah, Taif, Saudi Arabia; 3 Cancer Research UK & Medical Research Council Oxford Institute for Radiation Oncology, Department of Oncology, University of Oxford, Oxford, UK; 4 Diabetes Research Center, Qatar Biomedical Research Institute, Hamad Bin Khalifa University, Qatar Foundation, P.O. Box 34110 Doha, Qatar; 5 Proteomics Research Technology Platform, University of Warwick, University Hospital, Coventry, CV2 2DX UK

**Keywords:** Proteomics, Chaperones, Metabolic pathways, Diabetes complications

## Abstract

Metabolic dysfunction of endothelial cells in hyperglycemia contributes to the development of vascular complications of diabetes where increased reactive glycating agent, methylglyoxal (MG), is involved. We assessed if increased MG glycation induced proteotoxic stress, identifying related metabolic drivers and protein targets. Human aortal endothelial cells (HAECs) were incubated in high glucose concentration (20 mM versus 5 mM control) *in vitro* for 3–6 days. Flux of glucose metabolism, MG formation and glycation and changes in cytosolic protein abundances, MG modification and proteotoxic responses were assessed. Similar studies were performed with human microvascular endothelial HMEC-1 cells where similar outcomes were observed. HAECs exposed to high glucose concentration showed increased cellular concentration of MG (2.27 ± 0.21 versus 1.28 ± 0.03 pmol/10^6^ cells, P < 0.01) and formation of MG-modified proteins (24.0 ± 3.7 versus 14.1 ± 3.2 pmol/10^6^ cells/day; P < 0.001). In proteomics analysis, high glucose concentration increased proteins of the heat shock response – indicating activation of the unfolded protein response (UPR) with downstream inflammatory and pro-thrombotic responses. Proteins susceptible to MG modification were enriched in protein folding, protein synthesis, serine/threonine kinase signalling, glycolysis and gluconeogenesis. MG was increased in high glucose by increased flux of MG formation linked to increased glucose metabolism mediated by proteolytic stabilisation and increase of hexokinase-2 (HK-2); later potentiated by proteolytic down regulation of glyoxalase 1 (Glo1) - the major enzyme of MG metabolism. Silencing of Glo1, selectively increasing MG, activated the UPR similarly. Silencing of HK-2 prevented increased glucose metabolism and MG formation. *trans*-Resveratrol and hesperetin combination (tRES-HESP) corrected increased MG and glucose metabolism by increasing expression of Glo1 and decreasing expression of HK-2. Increased MG glycation activates the UPR in endothelial cells and thereby may contribute to endothelial cell dysfunction in diabetic vascular disease where tRES-HESP may provide effective therapy.

## Introduction

Increased plasma glucose concentration in diabetes induces dysfunction of endothelial cells (ECs) linked to development of diabetic vascular complications – nephropathy, retinopathy, peripheral neuropathy, generalised microangiopathy and increased risk of cardiovascular disease. This is characterized by increased inflammatory signalling, expression of adhesion molecules and secretion of inflammatory cytokines, apoptosis and processes supporting atherosclerosis^1–5^.

**Figure 1:**
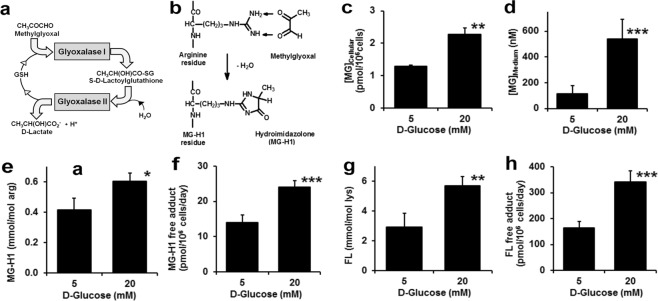
Dicarbonyl stress and increased protein glycation in human aortal endothelial cells in high glucose concentration. (**a**) The glyoxalase system. (**b**) Glycation of arginine residue by MG to form the major quantitative AGE, hydroimidazolone MG-H1. Dicarbonyl stress in HAECs after incubation for 6 days: (**c**) cellular MG, (**d**) MG concentration in conditioned culture medium, (**e**) MG-H1 adduct residue content of cytosolic protein, and (**f**) flux of MG-H1 free adduct released into culture medium. (**g**) FL adduct residue content of cytosolic protein, and (**h**) flux of FL free adduct released into culture medium. Data are mean ± SD (n = 3); significance: *, ** and ***P < 0.05, P < 0.01 and P < 0.001 with respect to low glucose concentration control; unpaired *t*-test.

An important contributor to EC dysfunction is accumulation of the reactive dicarbonyl metabolite, methylglyoxal (MG). MG is formed mainly by low-level degradation of triosephosphates and metabolised to D-lactate by the glyoxalase system. The glyoxalase system consists of two enzymes acting sequentially, glyoxalase 1 (Glo1) and glyoxalase 2 (Glo2), and a catalytic amount of reduced glutathione (Fig. 1a). Aldoketo reductase and aldehyde dehydrogenase may provide alternative minor pathways of MG metabolism. Abnormally increased MG concentration is called “dicarbonyl stress”. This may be driven by increased formation of MG, decreased activity of Glo1 and both of these metabolic changes combining together synergistically. MG is a potent glycating agent and precursor of the quantitatively major protein advanced glycation endproduct (AGE), arginine residue-derived hydroimidazolone MG-H1 (Fig. 1b). Overexpression of Glo1 prevented the development of vascular complications in experimental diabetes^6–9^. Decreased Glo1 expression was a driver of coronary heart disease in a large integrated transcriptomic translational study of non-diabetic and diabetic subjects^10^. Treatment of overweight and obese subjects with an optimised inducer of Glo1 expression - *trans*-resveratrol and hesperetin combination (tRES-HESP) - improved arterial dilatation and decreased vascular inflammation^11^.

Human aortal endothelial cells (HAECs) and the HMEC-1 microvascular endothelial cell line incubated in high glucose concentration have been employed as models of EC dysfunction in hyperglycemia^2,12^. When incubated in high glucose concentration, they have: increased cellular MG concentration and increased MG-H1 content of cell protein; increased production of inflammatory mediators, extracellular matrix proteins and adhesion molecules and apoptosis; and impaired angiogenesis. Many of these effects were prevented by overexpression of Glo1 and exacerbated by siRNA silencing and chemical inhibition of Glo1^4,5,12,13^.

It is currently unclear how dicarbonyl stress induces inflammatory and thrombotic responses in ECs. Our working hypothesis is that this occurs by MG-modified proteins being sensed as “damaged” and activating the cellular proteome quality surveillance system – the unfolded protein response (UPR). The aim of this study is to test this hypothesis and identify the metabolic drivers for increased MG concentration in ECs in high glucose concentration, proteins susceptible to glycation by MG and the pathways in which high glucose-induced changes of protein abundance and MG-modified proteins are enriched.

## Results

### Dicarbonyl stress in endothelial cells incubated in high glucose concentration and metabolic factors producing it

HAECs (seeding density 1.0 × 10^4^ cells/cm^2^) were cultured in 5 mM D-glucose and 20 mM D-glucose for 6 days. Cell viability remained high and viable cell number increased during this time, being unchanged by high glucose concentration (final cell density: 5.36 ± 0.67 and 5.24 ± 0.87 × 10^4^ cells/cm^2^, respectively; cell viability 98–99%, n = 6). HMEC-1 cells (seeding density 1.6 × 10^4^ cells/cm^2^) were cultured in 5 mM D-glucose and 30 mM D-glucose for 6 days. The growth rate was faster than of primary HAECs and was unchanged by high glucose concentration (final cell density: 27.9 ± 1.0 and 26.6 ± 1.7 × 10^4^ cells/cm^2^, respectively; cell viability 99–100%, n = 3). These findings are similar to previous studies^2,14^.

For HAECs incubated in high glucose concentration, the cellular content of MG was increased 2-fold and the concentration of MG in the culture medium was increased 5-fold, compared to low glucose concentration (5 mM) controls (Fig. 1c,d). There was a concomitant increase in MG modification of cellular protein: 45% increase in MG-H1 residue content of cytosolic protein extract (Fig. 1e) and 70% increase in flux of MG-H1 free adducts (MG-modified arginine) released into the culture medium (Fig. 1f). This indicates that HAECs suffer dicarbonyl stress in high glucose concentration leading to increased protein glycation by MG and increase in the steady-state level of MG-modified cellular protein. There was a similar proportionate increase of the glucose-derived early glycation adduct, N_ε_-fructosyl-lysine (FL), residues in cytosolic protein extracts and flux of FL free adduct released into the culture medium (Fig. 1g,h), indicating that HAECs suffer persistent high cytosolic glucose concentration in model hyperglycemia.

**Figure 2:**
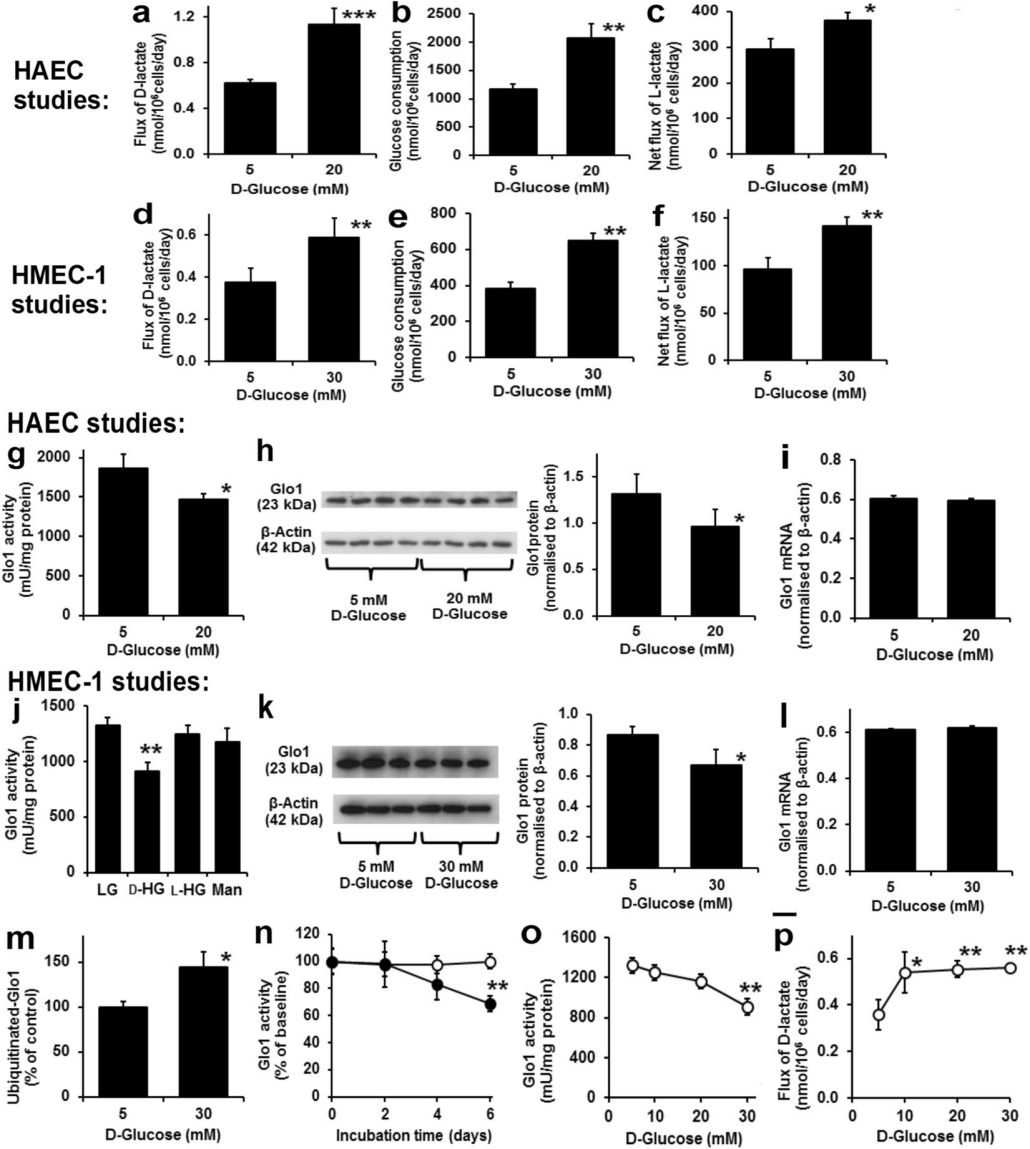
Metabolic drivers of dicarbonyl stress imposed by high glucose concentration in human endothelial cells in high glucose concentration *in vitro*. INCREASED MG FORMATION. HAECs: (**a**) Flux of D-lactate formation. (**b**) Glucose consumption. (**c**) Net flux of L-lactate formation. HMEC-1 cells. (**d**) Flux of D-lactate formation. (**e**) Glucose consumption. (**f**) Net flux of L-lactate formation. DOWN REGULATION OF GLO1. HAECs: (**g**) Glo1 activity. (**h**) Glo1 protein by Western blotting. (**i**) Glo1 mRNA. HMEC-1 cells: (**j**) Glo1 activity – effect of 5 mM D-glucose (LG), 30 mM D-glucose (D-HG), 5 mM D-glucose + 25 mM L-glucose (L-HG), and 5 mM D-glucose + 25 mM mannitol (Man). Significance: P = 0.042; *ANOVA*. (**k**) Glo1 protein by Western blotting. (**l**) Glo1 mRNA. (**m**) Glo1 ubiquitination. (**n**) Time course of Glo1 activity decline. Significance: treatment, P = 0.017; treatment by time, P < 0.001; *ANOVA repeated measures*. (**o**,**p**) Glucose concentration-dependent response of decreased Glo1 activity and increased flux of D-lactate, respectively. Significance: P = 0.001 and P = 0.008, respectively; *ANOVA*. Cell incubations were for 6 days unless otherwise stated. Data are mean ± SD (n = 3 except for n = 4 in (**h**)). Significance: *, ** and ***P < 0.05, P < 0.01 and P < 0.001, respectively, with respect to low glucose concentration control; *unpaired t-test* (unless otherwise stated). For metabolic flux measurements, analytes were determined at baseline and day 6 with the mean rate of change deduced.

We studied likely sources of increased MG concentration in HAECs: increased flux of MG formation and decreased enzymatic metabolism of MG. There was an 82% increase in the flux of D-lactate formation in incubations with high glucose concentration (Fig. 2a). Addition of exogenous D-lactate to HAEC cultures showed that the metabolism of D-lactate was very slow: 0.029 ± 0.005 nmol/day/million cells at D-lactate concentrations found in cultures with 5 mM glucose, equivalent to *ca*. 4% of the observed net rate of D-lactate formation. Therefore, the observed rate of D-lactate accumulation was an approximate measure of the flux of formation of MG^15^. There was similar increased flux of glucose consumption in high glucose concentration medium (+77%) (Fig. 2b). The flux of D-lactate formation, expressed as a percentage of flux of glucose consumption, was unchanged in high glucose concentration: 5 mM glucose, 0.053 ± 0.004% and 20 mM glucose, 0.055 ± 0.008%. The increased flux of formation of MG in high glucose concentration cultures was, therefore, that expected from increased flux of glucose metabolism through glycolysis. The net flux of formation of L-lactate was increased only 27% in high glucose concentration cultures (Fig. 2c), suggesting there was increased further metabolism of L-lactate via pyruvate in the tricarboxylic acid (TCA) cycle. A similar effect was observed in HMEC-1 cells, albeit achieved with a higher glucose concentration (30 mM) - Fig. 2d–f.

The activity of Glo1 in HAECs incubated in 5 mM D-glucose was 1862 ± 178 mU/mg protein. Other potential enzyme activities of MG metabolism, MG reductase and MG dehydrogenase, were undetectable under assay conditions (<26 mU/mg protein), indicating that Glo1 is the major pathway of MG metabolism in HAECs. Incubation of HAECs with 20 mM glucose for 6 days produced a downregulation of Glo1 activity and protein, decreasing 21% and 27%, respectively, without change in Glo1 mRNA (Fig. 2g–i). There was no change of activity of Glo2 – *data not shown*. A similar effect was observed in HMEC-1 cells where decrease of Glo1 was produced by incubation with high D-glucose concentration but not with non-metabolizable L-glucose nor mannitol – a control for increased osmolarity (Fig. 2j–l). We explored the role of proteolysis in change of Glo1 protein in high glucose concentration cultures by making ubiquitinated cell protein extracts from HMEC-1 cells by immunoaffinity pulldown and quantifying Glo1 protein. This indicated that the fraction of Glo1 ubiquitinated was increased in high glucose concentration cultures, consistent with the decrease in Glo1 activity and protein being mediated by increased proteolysis (Fig. 2m). In HMEC-1 cells, the decrease in Glo1 activity occurred late in the high glucose concentration culture, from days 4–6 and required exposure to higher glucose concentration than to increase flux of formation of D-lactate (Fig. 2n–p), suggesting that increased glucose metabolism and formation of MG precede decline in Glo1 protein on a time- and glucose concentration-dependent basis. Supporting this, increased MG content of HAEC and HMEC-1 cells in high glucose concentration cultures was previously found after incubation for 1–2 days without decrease in Glo1 activity^2,4^.

### Impact of high glucose on the cytosolic proteome - evidence for activation of the unfolded protein response by methylglyoxal

**Figure 3:**
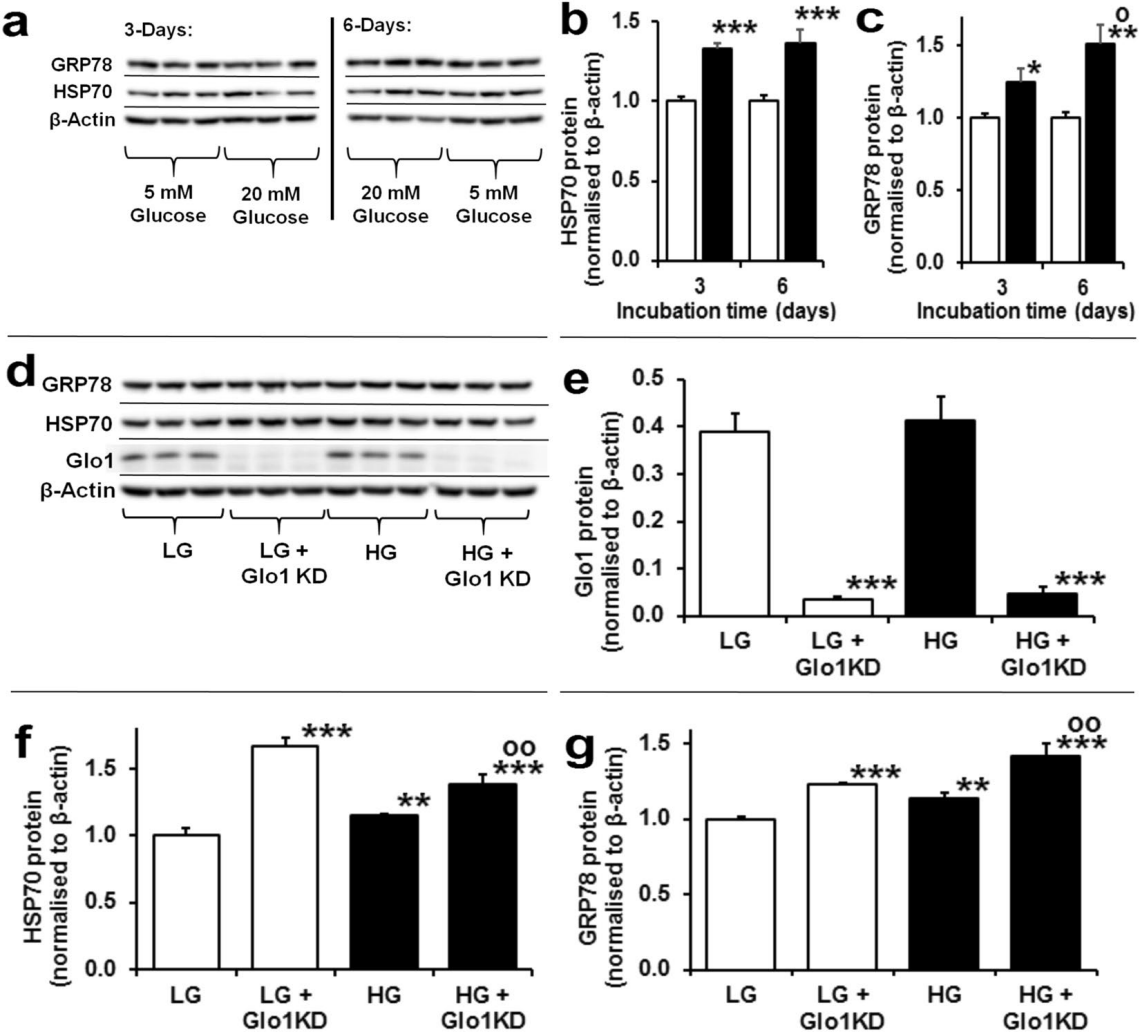
Activation of the unfolded protein response by dicarbonyl stress in human aortic endothelial cells in high glucose concentration. (**a**) Western blotting of heat shock protein response in HAECs incubated in 5 mM D-glucose (LG) and 20 mM D-glucose (HG) or 3 and 6 days. Western blot quantitation: (**b**) HSP70 (treatment, P = 0.012; *ANOVA repeated measures*); and (**c**) GRP78 (time, P = 0.015, treatment × time, P = 0.032; *ANOVA repeated measures*). (**d**) Western blotting of heat shock protein response in HAECs with siRNA knockdown of Glo1 incubated for 3 days under LG and HG conditions. Western blot quantitation: (**e**) Glo1 (P = 5 × 10^−7^; *ANOVA*), (**f**) HSP70 (P = 2 × 10^−6^; *ANOVA*) and (**g**) GRP78 (P = 3 × 10^−5^; *ANOVA*). Immunoblotting quantitation data are mean ± SD (n = 3). Key: empty bars, 5 mM D-glucose (LG); and filled bars, 20 mM D-glucose (HG). Significance: (**b**,**d**) *, ** and ***P < 0.5, P < 0.01 and P < 0.001 with respect to LG control, o, P < 0.05 with respect to 3 day HG control; (**e**) ***P < 0.001 with respect to scrambled siRNA transfected control (**f**,**g**) ** and ***P < 0.01 and P < 0.001 with respect to LG control, oo, P < 0.01 with respect to LG control; *unpaired t-test* (unless otherwise stated).

To gain insight into the impact of high glucose on cell function we investigated changes in abundance of cytosolic proteins of HAECs in high glucose concentration cultures by label-free quantitative proteomics. The mean number of proteins identified in low and high glucose concentration was 1894. There was 331 proteins upregulated in high glucose concentration (Supplementary Table 1). The protein abundance increases ranged from 8–254%. Proteins increased were enriched in the following pathways: gluconeogenesis, glycolysis, heat shock response – part of the UPR, separation of sister chromatids in mitosis, AU-rich elements/poly(U)-binding/degradation factor 1 regulated decay of mRNA and cytosolic tRNA aminoacylation (Table 1). We validated increased activity of the UPR in high glucose concentration cultures by immunoblotting of heat shock protein 70 kDa - 1A and 1B (HSP70) and glucose regulated protein-78 (GRP78) – markers of the cytosolic and endoplasmic reticulum UPR pathways. By immunoblotting, cellular HSP70 protein was increased 33% and 37% at day 3 and 6, respectively, and cellular GRP78 protein increased progressively from 25% and 51% at day 3 and 6, respectively (Fig. 3a–c). To assess the involvement of dicarbonyl stress on the induction of HSPs in high glucose concentration cultures, we analysed these UPR markers in HAECs with siRNA knockdown of Glo1 expression to 9–11% of control levels (Fig. 3d,e). Glo1 knockdown in HAECs was applied for 3 days to mimic the decreased Glo1 activity in the last 3 days of the 6-day cultures. Glo1 knockdown increased cellular MG concentration and increased cellular protein MG-H1 content of cellular protein^4^. It also increased HSP70 and GRP78 protein in low glucose concentration cultures and potentiated increase of HSP70 and GRP78 protein in high glucose concentration cultures (Fig. 3f,g), indicating that dicarbonyl stress is a driver of UPR activation.

**Table 1:** Pathways enrichment analysis of proteins in high glucose cultures.

Process	Pathway	Abundance Mean ± SD	Count	Fold enrichment	Significance	FDR	Proteins (Uniprot ID)
**Proteins with INCREASED abundance in high glucose concentration**							
Glucose metabolism	Gluconeogenesis	1.80 ± 0.54	8	8.8	1.1E-02	3.5E-02	ALDOA, G3P, MDHM, TPIS, PGAM1, AATC, ALDOC, PGK1
	Glycolysis	1.56 ± 0.28	11	12.1	4.8E-06	1.5E-05	ALDOA, PFKAP, KPYM, G3P, TPIS, HXK2, PP2AB, PGAM1, PP2AA, ALDOC, PGK1
Cell responses to stress	Regulation of HSF1-mediated heat shock response	1.36 ± 0.10	11	5.5	1.1E-02	3.5E-02	HSP7C, NU214, HS71B, RBP2, NU133, HS71L, HS105, BAG5, GRP78, RFA1, HS71A, GRP75
Cell cycle	Separation of Sister Chromatids	1.36 ± 0.20	18	3.4	8.5E-03	2.6E-02	PSMD2, NUDC, RAGP1, CLIP1, TBB4A, PSME3, PSD13, PP2AB, PSD12, PP2AA, TBB4B, PSMD3, RBP2, PSMD1, NU133, PRS6A, XPO1, PRS10
Metabolism of RNA	AUF1 (hnRNP D0) binds and destabilizes mRNA	1.34 ± 0.15	11	6.9	1.5E-03	4.5E-03	HSP7C, PSMD2, PSMD3, HS71B, PSME3, PSMD1, PRS6A, PSD13, PSD12, HS71A, HSPB1, PRS10
Metabolism of amino acids	SeMet incorporation into proteins	1.27 ± 0.10	9	28.8	2.6E-08	8.1E-08	SYRC, SYEP, SYMC, SYK, MCA3, AIMP1, SYLC, SYQ, SYDC
Protein synthesis	Cytosolic tRNA aminoacylation	1.27 ± 0.10	12	17.6	6.4E-09	2.0E-08	SYRC, SYEP, SYMC, SYK, SYHC, MCA3, AIMP1, SYCC, SYLC, SYQ, IPYR, SYDC
**Proteins with DECREASED abundance in high glucose concentration**							
Protein synthesis	L13a-mediated translational control	0.76 ± 0.08	6	16.3	3.1E-03	2.7E-02	RL5, RS2, RS20, RL35, RL13A, IF4E

Other proteins INCREASED in high glucose concentration (Protein, increase, function): PAI-1, +118%, fibrinolysis inhibitor; procollagen-lysine, 2-oxoglutarate 5-dioxygenase (PLOD), forms 1, 2 and 3, +52, +30% & +41%, post-translational processing of collagen; dihydropyrimidinase like 2, +38%, unknown function in ECs; fructose-2,6-bisphosphatase (TIGAR), +35%, increase of glycolysis; rho-associated protein kinase-1 (ROCK1), +20%, mitochondrial fission; ATG7, +19%, autophagy; BH3 interacting-domain death agonist (BID), +18, apoptosis. For pathways enrichment analysis, the significance given is with Bonferroni correction applied. All pathways with Bonferroni significance and FDR < 0.05 are shown, rank ordered by abundance increase (high to low).

There were 49 proteins decreased in the high glucose concentration cultures of HAECs (Supplementary Table 2), with protein abundance decreased by 7 to 81% of low glucose concentration control values. These proteins were enriched in the L13a-mediated translational control pathway (Table 1). Other key proteins downregulated were: annexin-A1, decreased 81% - a mediator of EC migration; annexin-A5, decreased 74% - a suppressor of EC thrombin formation; and chromobox protein homolog-5 (CBX5), decreased 30% - a regulator of EC progenitor differentiation and repression of vascular cell inflammation.

### Targets of methylglyoxal modification in the cytosolic proteome of endothelial cells

To gain insight into why dicarbonyl stress may be activating the UPR, we interrogated proteomics data for evidence of proteins modified by MG (+54 Da mass increment on arginine residues, reflecting MG-H1 formation). In high glucose concentration cultures of HAECs, only two proteins were detected with MG modification: rho GDP-dissociation inhibitor 2 (RhoGDI2) and far upstream element-binding protein 2 (FUBP2); others were below the limit of detection. For RhoGDI2, MG modification was detected on R148; and for FUBP2, MG modifications were located at R331 and R340.

**Figure 4:**
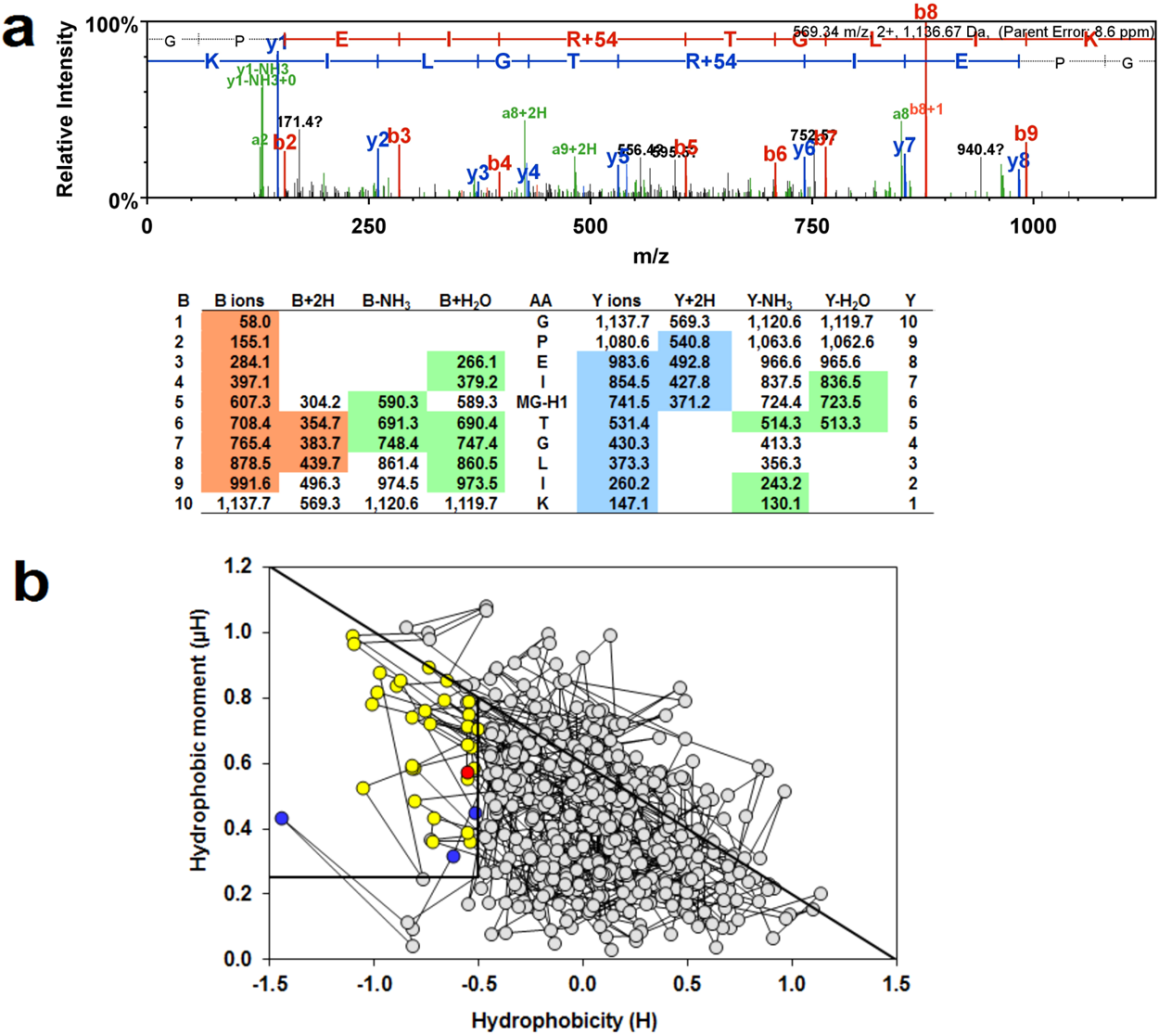
Mass spectrometric detection of methylglyoxal-modified proteins and prediction of functional impact by RBD analysis. (**a**) MG-modified peptide identification. Pyruvate kinase-M, R120. Peptide fragmentation ion mass scan of peptide 116–125 with MG-H modification on R120 (+54.01): GPEIR_MG-H1_TGLIK; with fragment ion assignment table. Ions detected are color coded in the mass spectrum assignments with color shading in the table. m/z ion values unshaded were not detected and blank spaces in the table indicate ions are not expected chemically. Unique peptide with: peptide identity probability 0.997, mascot ion score 39.7, observed mass (m/z) 569.33 (2+), actual peptide mass 1136.645 amu, calculated +1H peptide mass 1137.663 amu. “Parent mass error” indicates error on the peptide mass r. (**b**) RBD plot for human pyruvate kinase-M2. Line-linked filled circles represent the primary sequence. The RBD is the area bound by the trapezium in the upper left-side region of the chart. Key: circle with red fill, MG-H1 residue in the RBD; circles with blue fill, unmodified arginine residues in the RBD; circles with yellow fill, other amino acid residues in the RBD; and circles with grey fill, amino acid residues outside the RBD.

To explore other proteins susceptible to MG modification in dicarbonyl stress, we incubated HMEC-1 cytosolic protein extract with exogenous MG to increase the mean MG-H1 content by 10-fold – reflecting the upper limit of clinical dicarbonyl stress^16^. This was preferred over immunoaffinity enrichment of MG-modified proteins to avoid artefacts of antibody non-specific binding or antibody binding limited to selected MG-H1 peptide epitopes – as discussed^17,18^. The mean extent of modification by MG of arginine residues, however, is still very low: 0.04% in native samples and 0.4% in the cytosolic extract with 10-fold increased MG modification. Under these conditions, there were a total of 411 sites of MG-H1 modification detected on 220 proteins with 1–11 sites modified per protein molecule (Supplementary Table 3). A total of 1262 proteins were detected; therefore, 17% of proteins detected had low level modification by MG. Proteins with the highest number of modifications sites were: pyruvate kinase-M – 11 sites, and β-actin, α-enolase and heat shock protein 90-beta – 9 sites. An example of detection of MG-H1 modification is given for modified R120 in pyruvate kinase-M (Fig. 4a). Pathways analysis showed that MG-modified proteins were enriched in: protein folding, protein synthesis, glycolysis and gluconeogenesis (Supplementary Table 4). Protein domain targets of MG modification were: TCP-1 chaperonins, phosphoserine and phosphothreonine binding sites of 14-3-3 proteins, GroEL chaperonins, proteasome alpha/beta subunits and conserved sites of aminoacyl-tRNA synthases (Table 2). All have conserved functional arginine residues^19–23^. To assess if the site of MG modification found is likely associated with functional impairment, we deduced the proportion of the MG modification sites that are located in protein domains involved in functional interactions. We did this by receptor binding domain (RBD) analysis – a sequenced based bioinformatics approach to predict functional domains that is applicable to all proteins^24^. An example of an RBD plot for pyruvate kinase-M2 is given (Fig. 4b). In cytosolic proteins at risk of MG modification, 148 of the total 411 MG modification sites were in the RBD or 36%; and one or more MG modification sites in the RBD occurred in 104 of 220 proteins modified or 47%.

**Table 2:** Domain enrichment analysis of cytosolic proteins susceptible to glycation by methylglyoxal.

Process	Protein domain	Count	Fold enrichment
Protein folding	Chaperonin TCP-1, conserved site	7	68.1
	TCP-1-like chaperonin intermediate domain	7	51.1
	GroEL-like equatorial domain	7	40.9
	Chaperonin Cpn60/TCP-1	7	38.3
	GroEL-like apical domain	7	38.3
Signal transduction	14-3-3 domain	5	62.5
Proteolysis	Proteasome, subunit alpha/beta	8	36.9
Protein synthesis	Aminoacyl-tRNA synthetase, class II	6	30.9
Actin cytoskeleton	Actin/actin-like conserved site	7	29.2
	Actin, conserved site	6	29.2
	Actinin-type, actin-binding, conserved site	6	22.8
	Actin-related protein	7	18.0
Protein synthesis	Aminoacyl-tRNA synthetase, class I, conserved site	5	29.2
Nucleotide binding	Rossmann-like alpha/beta/alpha sandwich fold	7	14.6

Significant: P < 0.05 after Bonferroni correction and FDR < 0.05.

### Switch to hexokinase-2-dependent glucose metabolism increases glucose consumption by endothelial cells in high glucose

**Figure 5:**
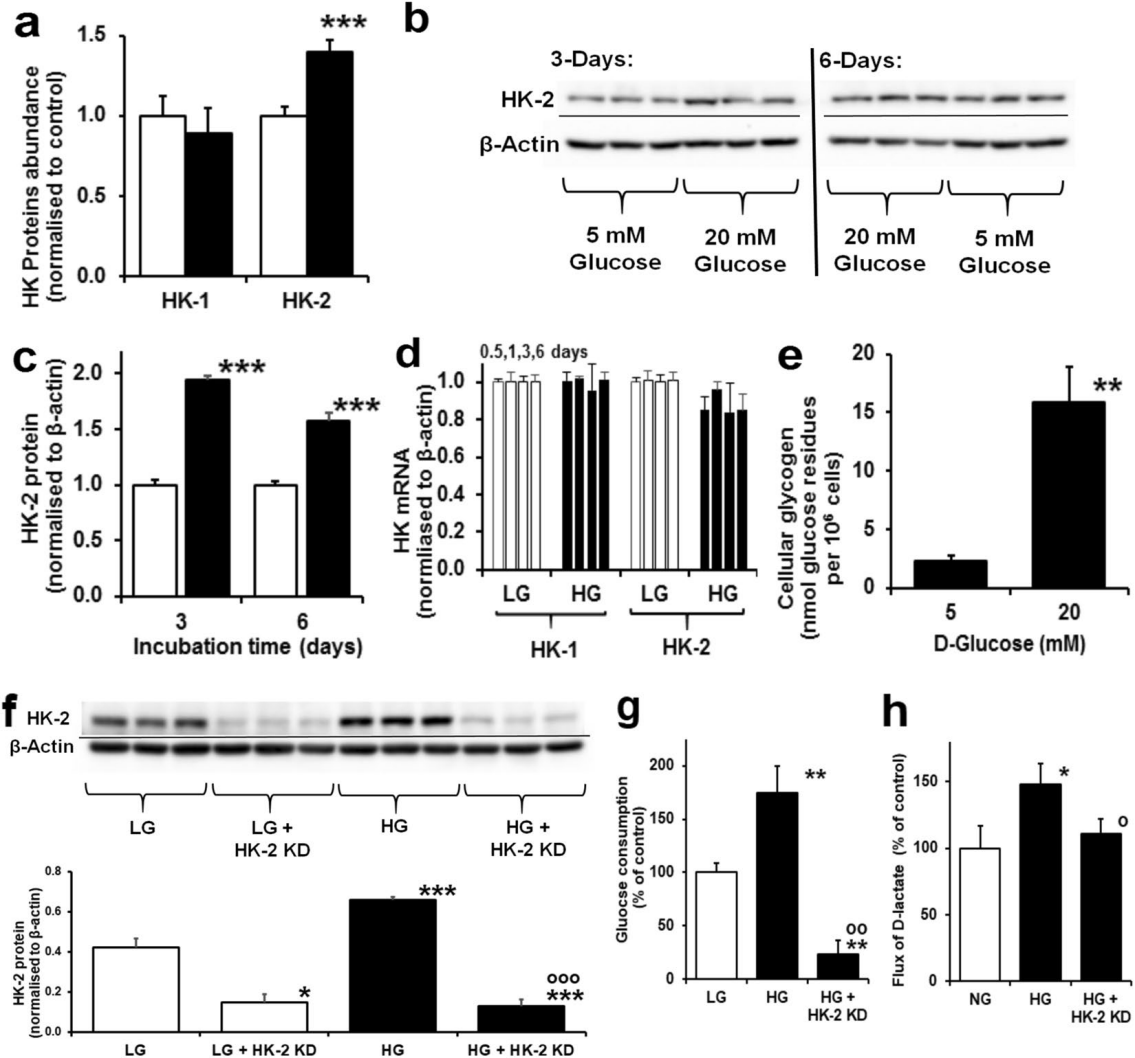
Increased hexokinase-2 drives increased glucose metabolism in human aortic endothelial cells in high glucose concentration. (**a**) HK-1 and HK-2 protein abundance by proteomics (normalised to 5 mM glucose control). (**b**) Western blotting of HK-2 protein. (**c**) Densitometry quantitation of HK-2 protein (time, P = 0.041, treatment, P = 0.003, time × treatment, P = 0.003; *ANOVA repeated measures*). (**d**) HK-1 and HK-2 mRNA at 0.5, 1, 3 and 6 days - columns from left to right (normalised to 5 mM glucose control). (**e**) HAEC glycogen content. Cell incubations were for 6 days unless otherwise stated. HK-2 gene silencing study: (**f**) Western blotting of HK-2 protein and densitometry quantitation of HK-2 protein, P < 0.001, *ANOVA*; (**g**) effect on glucose consumption, P < 0.001, *ANOVA*; and (**h**) effect on D-lactate formation, P = 0.003, *ANOVA*. Cell incubations were for 3 days. Data are mean ± SD (n = 3). Significance: *, ** and ***P < 0.05, P < 0.01 and P < 0.001 with respect to low glucose concentration control and o, oo and ooo, P < 0.05, P < 0.01 and P < 0.001 with respect to high glucose concentration control; unpaired *t*-test (unless otherwise stated).

To understand the mechanism of increased glucose consumption of ECs in high glucose concentration, we studied the abundances of hexokinase isoforms, hexokinase-1 (HK-1) and hexokinase-2 (HK-2) by label-free quantitative proteomics analysis of cytosolic protein extracts of HAECs. The abundance of HK-1 was unchanged in high glucose concentration cultures whereas HK-2 abundance was increased 40% (P < 0.05) (Fig. 5a). This was corroborated and confirmed by Western blotting (Fig. 5b,c). There was no increase of HK-2 mRNA (nor HK-1 mRNA) throughout the culture period (Fig. 5d), suggesting that HK-2 protein is selectively stabilised from proteolysis in cultures with high glucose concentration. In high glucose concentration conditions with increased total hexokinase activity, HK-1 remains strongly bound to mitochondria whereas HK-2 translocates between mitochondria and the cytosol in response to increased glucose-6-phosphate (G6P) concentration. A marker of loss of HK-2 from mitochondria to the cytosol is increased glycogen synthesis through metabolic channelling when in the cytosol^25^. Herein we found the glycogen content of HAECs increased 7-fold with 20 mM glucose (Fig. 5e). High glucose concentration therefore drives increased glucose consumption, glycolysis and MG formation through glucose-induced stabilisation of HK-2 with related increased formation of glycogen indicative of non-mitochondrial HK-2. To confirm this role, we studied the effect of knockdown of HK-2 on glucose metabolism and MG formation in HAECs in high glucose concentration. HK-2 Knockdown prevented high glucose concentration-induced increased consumption of glucose and MG formation, supporting a key role of increased HK-2 in metabolic dysfunction of ECs in model hyperglycemia (Fig. 5f–h).

### Reversal of dicarbonyl stress and increased glucose metabolism of human aortal endothelial cells in high glucose by tRES-HESP

**Figure 6:**
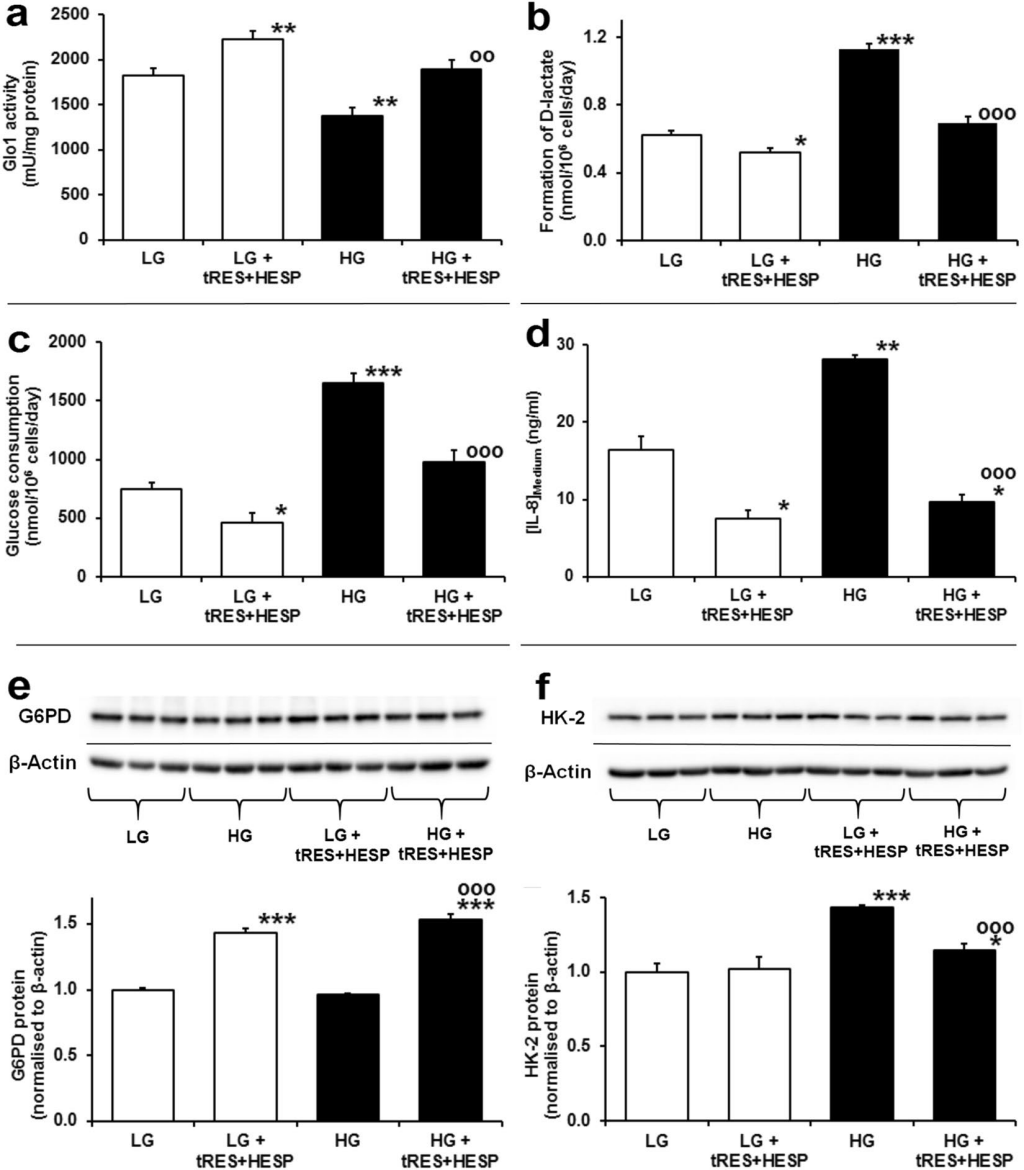
Reversal of metabolic dysfunction in human aortic endothelial cells in high glucose concentration by *trans*-resveratrol-hesperetin combination. Metabolic measurements. (**a**) Glo1 activity (P = 7 × 10^−11^, *ANOVA*). (**b**) Flux of D-lactate formation (P = 7 × 10^−11^, *ANOVA*). (**c**) Glucose consumption (P = 8 × 10^−9^, *ANOVA*). (**d**) Concentration of IL-8 in conditioned medium (P = 2 × 10^−5^, *ANOVA*). Western blotting. (**e**) G6PD protein (P = 1 × 10^−8^, *ANOVA*). (**f**) HK-2 protein (P = 4 × 10^−5^, *ANOVA*). Key: hollow bars and “LG”, incubations with 5 mM D-glucose; filled bars and “HG”, incubations with 20 mM D-glucose; and “tRES-HESP”, incubations with 10 μM tRES-HESP. Data are mean ± SD (n = 4 for (**a**); for (**c**–**f**) n = 3 except n = 8 for LG and HG controls in (**b**,**c**). Significance: *, ** and ***P < 0.05, P < 0.01 and P < 0.001 with respect to LG control; oo and ooo, P < 0.01 and P < 0.001 with respect to HG control (*unpaired t-test)*.

We finally considered if dicarbonyl stress and increased glucose consumption of HAECs in high glucose could be reversed by small molecule therapeutic agents. An effective intervention to suppress dicarbonyl stress is to increase the expression and activity of Glo1 by tRES-HESP^11^. Incubation of HAECs with 10 μM tRES-HESP increased Glo1 activity by 22% in low glucose concentration and prevented the decreased Glo1 activity in high glucose concentration (Fig. 6a). Surprisingly, tRES-HESP decreased the flux of MG formation by 17% in low glucose concentration and prevented the increased flux of MG formation in high glucose concentration, as judged by D-lactate formation (Fig. 6b). Remarkably, tRES-HESP also decreased glucose consumption by 38% in low glucose concentration and prevented the increase of glucose consumption in high glucose concentration (Fig. 6c), indicating that both the deficit of Glo1 activity and increased glucose consumption were corrected. Functional benefit of this was judged by assessing the levels of inflammatory mediator interleukin-8 (IL-8) which were decreased by tRES-HESP in low and high glucose concentration cultures (Fig. 6d).

Insight into the mechanism of reversal of increased glucose metabolism was gained from studies of glucose-6-phosphate dehydrogenase (G6PD) and HK-2 expression in HAECs. Expression of G6PD is increased by activation of Nrf2 by tRES-HESP in endothelial cells^26^. Herein, we found tRES-HESP induced increased expression of G6PD in high glucose concentration cultures (Fig. 6e). Related increased G6PD activity decreases steady-state levels of G6P and thereby suppresses Mondo A:Mlx functional activity dependent on availability of G6P cofactor^27^. Expression of HK-2 is regulated by Mondo A:Mlx^28^. Therefore, decrease of Mondo A:Mlx functional activity by tRES-HESP corrected the increased abundance of HK-2 in HAECs in high glucose concentration, with no effect in low glucose concentration likely due to basal HK-2 expression under these conditions (Fig. 6f).

## Discussion

Herein we show that ECs suffer dicarbonyl stress in high glucose concentration associated with increased MG formation synergising with decreased Glo1 activity and this activates the UPR with downstream development of a pro-inflammatory and pro-thrombotic phenotype.

**Figure 7:**
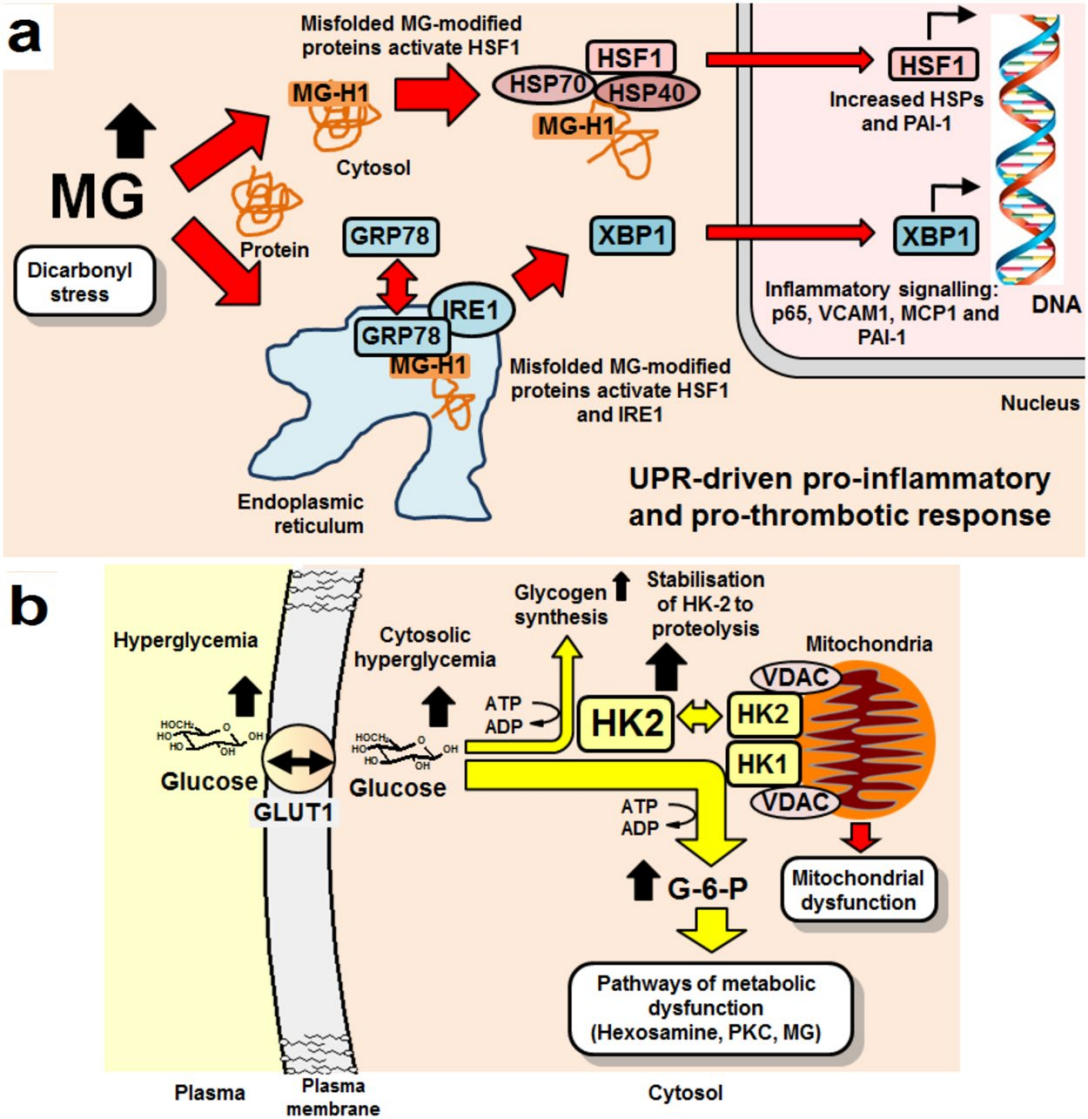
Schematic diagram of the mechanisms of activation of the unfolded protein responsive and pro-inflammatory response by dicarbonyl stress and increased glucose metabolism in endothelial cells in hyperglycemia. (**a**) Activation of the cytosolic and endoplasmic reticulum UPR by misfolded MG-modified proteins. (**b**) Increased glucose metabolism, mitochondrial dysfunction and glycogen synthesis by stabilisation of HK-2 to proteolysis by cytosolic hyperglycemia. Abbreviations: PKC, protein kinase C; VDAC, voltage-dependent anion channel.

A major finding was increased abundance of HSPs in high glucose concentration cultures of HAECs and induction of these by dicarbonyl stress. Heat shock cognate 70 (HSPA8) and HSP70 1A and 1B, 70 kDa protein 1-like heat shock protein, HSP 105 kDa, and 75 kDa and 78 kDa glucose-regulated proteins (HSPA9 and HSPA5) were increased in high glucose concentration cultures. Increased expression of HSPs of the HSF-1 pathway, part of the UPR^29^, may be due to release of HSF-1 from complexation with HSP70 and HSP40 by binding of increased MG-modified misfolded proteins, migration of HSF-1 to the nucleus and increased transcriptional activity for HSPs. MG-modified proteins are thereby likely funneled through the HSP pathway for degradation by proteasomal proteolysis and chaperone-mediated autophagy^29^. Hence, many MG-modified proteins are focused towards HSPs with subsequent activation of pro-inflammatory signaling (Fig. 7a).

GRP78 is usually localized to the endoplasmic reticulum. It was increased in the cytosolic fraction of HAECs in high glucose concentration cultures. Cytosolic translocation of GRP78 may be induced by MG-modified misfolded proteins and drives an autophagic response^30^, accounting for increase of the ATG7 protein in high glucose concentration cultures. Loss of GRP78 from complexation of inositol-requiring protein-1 drives activation downstream of endoplasmic reticulum stress transcription factor, X box-binding protein 1 (XBP1)^31^ and increased expression of histone H3 lysine 4 methyltransferase SET7^32^. SET7 expression is increased in HAECs incubated in high glucose concentration where MG-modified proteins are likely upstream inducers^4,33^. This has been linked to increased expression of MCP1, the receptor for AGEs (RAGE) and its ligands^12,33^. GRP78 also activates the NF-kB system by promoting the degradation of IkBα, with downstream up-regulation of ICAM-1, VCAM-1 and MCP-1^34^ (Fig. 7a).

A pro-thrombotic response was also detected in the proteomics studies where increased abundance of PAI-1 and decreased abundance of thrombin suppressor, annexin-A5, was found in HAECs in high glucose. Increased expression of PAI-1 in ECs is driven by activation of HSF-1 and NF-κB^35,36^ and these pathways converge and likely synergize in the activation of the UPR by dicarbonyl stress (Fig. 7b).

Inflammatory and pro-thrombotic signalling may be further exacerbated by MG modification of FUBP2. FUBP2 destabilizes mRNAs of inflammatory genes – including IL-8 and PAI-1^37^, and is considered a checkpoint for inflammatory cytokines. MG modification of FUBP2 may impair its functional activity and increase inflammatory signaling. The L13a-mediated translational control pathway was also down-regulated in high glucose concentration cultures which may contribute to increased inflammatory gene translation and pro-atherogenic response^38^. RhoGDI2 was also modified by MG on R148 in the geranylgeranyl-binding pocket that interacts with Rho GTPases^39^. This is likely linked to inhibition of RhoGDI and activation of Rac2 and endothelial NADPH oxidase^40^, increasing formation of reactive oxygen species (ROS).

We also characterised, for the first time, proteins of the EC cytosolic proteome susceptible to modification by MG. The potential impact of dicarbonyl stress on cell function was indicated by protein pathway and domain enrichment analysis. Key pathways enriched for MG modification were protein folding, protein synthesis, glycolysis and gluconeogenesis. Protein domains enriched with MG modification were Tailless Complex Polypeptide-1 (TCP-1) and GroEL protein domains of chaperonins. This suggests dicarbonyl stress preferentially impairs protein folding and may be why dicarbonyl stress activates the UPR since MG damages chaperonins – the “guardians” of correct protein folding. Phosphoserine and phosphothreonine binding sites of 14-3-3 proteins and proteasome alpha/beta subunits were also hotspots for MG modification, suggesting signalling by serine/threonine kinases and proteasomal proteolysis may be impaired in dicarbonyl stress. Impaired proteasome function has indeed been found in experimental diabetic vascular disease^41^. Functional impact also depends on whether modifications by MG occur within domains associated with protein functional activity. RBD analysis suggested that MG modification had a 36% probability of location in a site of protein functional interaction and thereby often likely causes protein inactivation or dysfunction. A similar probability, 43%, is found for application of RBD analysis to total arginine residues proteome-wide – an enrichment therein of 3.8 fold and highest of any amino acid. We conclude that MG modification is damaging, therefore, because it produces target inactivation through loss of charge by MG-H1 formation and its target, arginine residues, has a high probability of location in a protein functional domain. In contrast, FL adducts retain the lysine residue charge and have lower enrichment, 2.1 fold, in functional domains^24^.

In exploring factors that produce dicarbonyl stress in ECs incubated in high glucose concentration, we found that there was increased flux of MG formation concomitant with increased glucose metabolism. The rate of glucose metabolism by HAECs is not limited by glucose uptake: ECs exhibit GLUT1 glucose transporter-mediated glucose uptake which is rapid compared to the rate of entry of glucose into glycolysis catalysed by hexokinase. Consistent with this we observed increased FL residue content of HAEC protein, reflecting increased cytosolic glucose concentration in high glucose cultures. Rather, glucose metabolism in ECs is rate-limited by HK-1 and HK-2 which both operate under saturation kinetics in both normal and high glucose concentration conditions^42^. It was hitherto unclear how increased glucose metabolism occurred in ECs in high glucose cultures. HK-2, unlike HK-1, is degraded by chaperone-mediated autophagy where heat shock cognate 71 kDa protein binds to motif _712_QRFEK_716_. This motif is directly involved in the binding of glucose at the active site in the C-terminal domain. In the presence of high cytosolic glucose concentration, increased binding of glucose masks the degradation motif and HK-2 protein is stabilised to proteolysis^43^. This provides an explanation for increased flux of glucose metabolism under conditions of saturating glucose concentration of HK-1 and HK-2. From relative quantitation by proteomics and k_cat_ values (k_cat,HK-2_/k_cat,HK-1_ ≈ 5)^42^, we deduce that under normal glucose concentration conditions HK-2 represents 22% total HK protein and 59% HK activity. In high glucose concentration, HK-2 protein was increased by 94% (Fig. 5c) and total hexokinase activity by *ca*. 60%. Hence, this is the major mechanism driving the observed increased glucose metabolism of HAECs in high glucose^44^. This is consistent with increased concentration of G6P in HAECs in high glucose concentration^44^. Increased G6P partially displaces HK-2 from mitochondria, impairing mitochondrial oxygen consumption similar to that found under conditions of inhibition of ADP recycling^25^. This increases the mitochondrial membrane potential and ROS formation^45^, contributing to previously observed mitochondrial dysfunction^2,3^.

HK-2 in the cytosol drives metabolic channelling of G6P to glycogen synthesis. We found a 7-fold increase in glycogen content of HAECs incubated in high glucose concentration, although the net flux of glycogen formation was <1% of the increased glucose consumption. Increased glycogen deposition is, therefore, a highly sensitive biomarker of cellular metabolic dysfunction in high glucose. Abnormal glycogen deposition was found in renal tubular cells in clinical diabetic nephropathy^46^, retinal neurons and Muller cells in experimental diabetic retinopathy^47^, associated with demyelination and axonal degeneration in clinical diabetic neuropathy^48^ and in arteries of streptozotocin-induced diabetic rats^49^. From this finding, criteria for tissue susceptibility to metabolic dysfunction in hyperglycemia are high cytosolic glucose concentration – supported by GLUT1-mediated glucose transport^50^, and HK-2 expression. This explains susceptibility to hyperglycemia-induced metabolic dysfunction in the vasculature, kidney, retina and peripheral nerve in diabetes; and why impact on the brain is limited where HK-2 is undetectable^51^.

Increased glucose metabolism of ECs in high glucose provides metabolic flux for mitochondrial dysfunction, activation of protein kinase C and hexosamine pathways^2,3^ – as well as increased flux of MG formation (Fig. 7b). Increased glycolytic flux of HAECs in high glucose concentration is consistent with increased abundance of glycolytic enzymes in proteomics analysis. Increased enzymes of gluconeogenesis, malate dehydrogenase and aspartate aminotransferase, may represent a cataplerotic response to balance the anaplerotic effect of increased oxaloacetate entering the TCA cycle driven by increased L-lactate and pyruvate formation from increased glycolysis.

In reversing metabolic dysfunction of HAECs in high glucose concentration by tRES-HESP, we expected to find increased Glo1 activity as the main pharmacological response since tRES-HESP was optimized to induce Glo1 expression via activation of transcription factor Nrf2^11^. We also found that tRES-HESP reversed the increase of HK-2 protein in high glucose concentration cultures – which we attribute to induction of G6PD expression via activation of Nrf2^26^ and decrease of G6P, cofactor for Mondo A:Mlx complex regulation of HK-2 expression^27,28^. By suppressing HK-2 expression and increasing Glo1 expression, tRES-HESP cuts off the drivers of endothelial dysfunction in high glucose concentration at source.

## Conclusions

Increased MG concentrations of ECs incubated in high glucose concentration is induced by both increased MG formation and decreased metabolism. This increases MG protein glycation which is sensed by the UPR as a proteotoxic challenge with subsequent downstream development of a pro-inflammatory and pro-thrombotic phenotype. Increased glucose metabolism sustaining this response is produced by stabilisation of HK-2 to proteolysis in high cytosolic glucose concentration. tRES-HESP cuts off drivers of dicarbonyl stress at source and may provide effective treatment of endothelial cell dysfunction in diabetes.

## Methods

### Cell culture and reagents

HAECs were purchased was from Caltag Medsystems (Buckingham, U.K.; Cat# SC-6100) and human microvascular endothelial cell HMEC-1 line was from CDC (Atlanta, Georgia, USA). HAECs were cultured under an atmosphere of air with 5% CO_2_, 100% humidity, at 37 °C in human large vessel endothelial cell growth medium with growth supplement and antibiotic supplement (Cat# ZHM-2953) according to the manufacturer’s instructions. They were used during passages 4–6 which maintains the primary endothelial phenotype. The HMEC-1 cell line was cultured in MCDB-131 medium supplemented with 10% fetal bovine serum^2^. Cell viability was assessed by the Trypan blue exclusion method. For metabolic flux measurements, analytes were determined at baseline and day 6 with the mean rate of change deduced. Culture conditions were: low glucose concentration (5 mM D-glucose) and high glucose concentration (10, 20 and 30 mM D-glucose) with and without 10 µM tRES-HESP, 5 mM D-glucose with 25 mM L-glucose or 25 mM mannitol for 6 days. For Glo1 and knockdown studies, 2–8 × 10^5^ of HAECs were transfected with 5 nM Accell Human GLO1 SMART siRNA pool or an Accell non-targeting Control siRNA pool with Lipofectamine® RNAiMAX Transfection Reagent. After 24 h, the cells were treated with 5 mM or 20 mM glucose for 72 h. RNA and protein were then extracted and stored at −80 °C until further analysis. Hexokinase-2 (HK-2) expression was knocked down similarly. Other materials are listed in Supplementary Data. All methods were carried out in accordance with relevant guidelines and regulations and all experimental protocols were approved by University of Warwick Genetic Modification & Biosafety Committee (Project no. 305).

### Biochemical measurements

Glucose concentration in culture medium was assayed by the hexokinase method. L-Lactate and D-lactate concentrations in culture medium were assayed by endpoint enzymatic assay and activity of Glo1, MG reductase and MG dehydrogenase were assayed as described^52^. MG content of HAEC cells and culture medium, and MG-H1 and glucose-derived N_ε_-fructosyl-lysine (FL) protein glycation adduct residues in cytosolic protein extracts and related free adducts in culture medium were determined by stable isotopic dilution analysis liquid chromatography-tandem mass spectrometry^53,54^. IL-8 concentration of culture medium and cellular glycogen content were measured with commercial ELISA kits.

Real-time PCR and Western-blotting was performed as previously described^55^. The primers for each gene are given in Supplementary Table 5. The reverse transcriptase reaction was performed total RNA (100 ng, 20 µl) using High-Capacity cDNA Reverse Transcription Kit (Applied Biosystems™) and run on an Eppendorf Mastercycler gradient. The reaction was incubated at 25 °C for 10 min, then 37 °C for 2 h, and then 85 °C for 5 min. After 5 fold dilution, 2 µl reverse transcription product cDNA was used for qRT-PCR to detect each target gene expression level using SYBR Green technique with SYBR^®^ qPCR ReadyMix™ Low ROX™ kit on a ABI 7500 real time PCR system in 20 µl reaction volume. The reaction started at 95 °C for 2 min and followed 40 cycles at 95 °C for 15 s and 60 °C for 1 min. The relative quantification for each gene expression level was evaluated using 2^**(−**ddCt**)**^, and data were normalized by ACTB as a reference gene. Assay reactions were performed in triplicate.

For Western blotting, cell protein was prepared with RIPA buffer with protease and phosphatase inhibitor cocktail. The DC protein assay kit was used to determine protein concentration. Cell protein extracts (30 µg) were loaded to SDS/PAGE 10% polyacrylamide gels. After electrophoresis, the proteins were transferred to a PVDF membrane and the membrane was blocked with 10% (w/v) non-fat dried skimmed milk powder in Tris-buffered saline (TBST; 10 mM Tris/HCl, pH 7.5, 150 mM NaCl and 0.05% Tween 20). The membrane was incubated with primary antibodies at 4 ◦C overnight. After washing with TBST, the membrane incubated with appropriate secondary antibody-horseradish peroxidase conjugate for 1 h at room temperature. Immunoreactivity was detected using enhanced chemiluminescence (ECL) and visualized with GNOME XRQ NPC chemiluminescence imaging (Syngene). The intensities of protein bands were quantified by software ImageQuant TL (GE Healthcare). For the reference protein β-actin, the membrane was stripped with stripping buffer (100 mM 2-mercaptoethanol, 2% SDS and 62.5 mM Tris/HCl, pH 6.8), blocked with 5% (w/v) non-fat dried skimmed milk powder in TBST and re-probed with anti-β-actin antibody with ECL detection. Antibody dilution used as recommended by the supplier or as indicated previously for Glo1 antibody prepared in-house^56^.

### Proteomics and bioinformatics analysis

Cytosolic protein extracts were analysed for MG-H1-modified proteins by high resolution Orbitrap mass spectrometry of tryptic digests, as described^17^. Cytosolic protein extracts were prepared form HAECs incubated for 6 days with 5 mM and 20 mM D-glucose and cytosolic protein extract of HMEC-1 cells incubated with 500 µM MG for 24 h at 37 °C increasing the MG-H1 residue content by 10-fold to 5 mmol/mol arg. Reduced and alkylated protein extracts were digested with Lys-C and TPCK-treated trypsin. Mean sequence coverage for proteins identified in HAECs was 22.4 ± 0.8% (5 mM glucose) and 22.6 ± 0.2% (20 mM glucose). Label free quantitation of protein abundances were determined in three independent biological replicate samples using Progenesis QI for proteomics 2.0 software (Nonlinear Dynamics, Newcastle, UK). Protein ontology was evaluated using the Database for Annotation, Visualization and Integrated Discovery v6.8 (https://david.ncifcrf.gov/)^57^ to identify molecular functions and biologic processes that may be impacted by changes in protein abundance and MG modification. REACTOME and INTERPRO analysis^24^ was used for pathway and protein domain enrichment analysis. To identify if MG modifications target functional domains of proteins, we identified functional domains by sequence-based receptor binding domain (RBD) analysis applied proteome-wide and deduced the number of MG modifications in functional and non-functional domains - see Supplementary Methods.

### Statistical analysis

Data are mean ± SD or SEM of ≥3 independent biological replicates and responses were validated for HAECs in cells from 3 different donors. Test samples were analysed randomly. Significance of difference of two groups by Student’s *t-test* and of >2 groups by ANOVA or ANOVA repeated measures (with Bonferroni correction).

## Supplementary information

Supplementary information, tables and original gel images
