# Supplementary material for: Activation of the unfolded protein response in high glucose treated endothelial cells is mediated by methylglyoxal
Source: Sci Rep. 2019 May 27;9:7889. doi: 10.1038/s41598-019-44358-1 (PMC6536510; doi:10.1038/s41598-019-44358-1)

Supplementary information, tables and original gel images

Activation of the unfolded protein response in high glucose treated endothelial cells is mediated by methylglyoxal

Zehra Irshad,[1]* Mingzhan Xue,[1]* Amal Ashour,[1,2] James R. Larkin,[1,3] Paul J. Thornalley[1,4] and Naila Rabbani[1,5]

Materials L-Lactic dehydrogenase from bovine heart, type III (Cat# L2625), D-lactic dehydrogenase from *Staphylococcus epidermidis* (Cat# L9636), L-lactate, sodium salt (Cat# 71718), D-lactate, sodium salt (Cat# 71716), 1,2-diaminobenzene, sublimed (Cat# 694975), *trans*-resveratrol (Cat# R5010), hesperetin (Cat# W431300), pepsin from porcine stomach mucosa (Cat# P6887), prolidase from porcine kidney (Cat# P6675), pronase E - type XIV from *Streptomyces griseus* (Cat# P5147), leucine aminopeptidase - type VI from porcine kidney (Cat# L6007), DL-dithiothreitol (Cat# 43819), iodoacetamide (Cat# I1149), trypsin, N-*p*-tosyl-L-phenylalanine chloromethyl ketone-treated (Cat# 4352157-1KT), endoproteinase Lys-C - sequencing grade from *Lysobacter enzymogenes* (Cat# 11047825001) and KiCqStart® SYBR® Green qPCR ReadyMix™ real time quantitative PCR kit (Cat# KCQS01) were from Sigma-Aldrich (Poole, Dorset, U.K.). Radio-immunoprecipitation assay (RIPA) buffer (Cat# 9806) was from Cell Signaling Technology (Leiden, The Netherlands). Antibodies: hexokinase-2 antibody (Cat# ab131196), HSP70 antibody (Cat# ab194360), glucose-6-phosphate dehydrogenase antibody (Cat# ab124738) and β-actin antibody (Cat# ab8229) were from Abcam (Cambridge, U.K.); rabbit IgG antibody (Cat# A9169) was from Sigma-Aldrich; and GRP 78 antibody - A-10 (Cat# sc-376768) was from Santa Cruz Biotechnology **(**Heidelberg, Germany). Commercial assay kits: glucose assay - hexokinase method (Cat# GAHK2) was from Sigma-Aldrich; glycogen assay kit II– colorimetric (Cat#

ab169558) was from Abcam; ubiquitination protein enrichment kit (Cat# 662200) was from Merck Ltd (Watford, U.K.); interleukin-8 immunoassay kit - Quantikine@ELISA (Cat# D8000C) was from R & D Systems (Abingdon, U.K.); and DC protein assay kit II (Cat# 5000112) was from Bio-Rad (Watford, U.K.).

Supplementary methods

Proteomics and bioinformatics analysis Cytosolic protein extracts were prepared form HAECs incubated for 6 days with 5 mM and 20 mM D-glucose and cytosolic protein extract of HMEC-1 cells that had been incubated with 500 µM MG for 24 h at 37 ^{o}C increasing the MG-H1 residue content by 10-fold to 5.0 mmol/mol arg. Cell pellets were suspended in aliquots of 10 mM sodium phosphate buffer, pH 7.0 (400 µl) and sonicated on ice (100 W, 30 s). Samples were centrifuged (20,000 g, 30 min, 4°C) to sediment membranes. The supernatant was transferred and stored at -80°C until analysis. Lysate (500 µg) was washed by 4 cycles of concentration to 40 µl and dilution to 400 µl with water over microspin ultrafilter (10 kDa cut-off) at 4°C. The final washed protein concentration was determined by Bradford method. An aliquot of cytosolic protein extract (100 µg, 50 µl) was treated with dithiothreitol (6 µl, 6 mM) and incubated at 37°C in the dark for 30 min; and then treated with iodoacetamide (5.9 µl, 10.8 mM) and incubated at 37°C in the dark for 30 min. Residual iodoacetamide was then quenched by further addition of dithiothreitol (5.9 µl 6 mM) and incubated at 37°C in the dark for 30 min. An aliquot of Lys-C protease (1 mg/ml, 5 µl) in 500 mM ammonium bicarbonate, pH 8.0, was added and incubated for 1 h at 37°C. Then TPCK-treated trypsin (1 mg/ml, 5 µl) in 1 mM calcium chloride/500 mM ammonium bicarbonate, pH 8.0, was added and samples were incubated at 37°C for 5 h in the dark. Finally, the reaction was stopped by adding 10% TFA (5 µl) in water. The sample was lyophilised to

dryness to remove volatile salts and re-suspend in an aliquot (100µl) 0.1% formic acid in water and analysed by nanoflow liquid-chromatography-Orbitrap mass spectrometry.

The processed cell lysate samples were submitted to the Mass Spectrometry and Proteomics Facility at Warwick University for a label-free proteomic quantitation analysis. Reversed phase nanoflow liquid chromatography-mass spectrometry for global protein identification was performed on an Orbitrap mass spectrometer (Fusion™ Tribrid™, ThermoFisher Scientific) equipped with a microspray source operating in positive ion mode. For proteomics analysis the column used was: an Acclaim PepMap µ-pre-column cartridge (trap), 300 µm i.d. x 5 mm, 5 µm particle size, 100 Å pore size, fitted to an Acclaim PepMap RSLC 75 µm i.d. x 50 cm, 2 µm particle size, 100 Å pore size main column (ThermoFisher Scientific). It was installed on an Ultimate 3000 RSLCnano system (Dionex). An aliquot (5 µl) of sample was injected. After injection, the peptides were eluted off of the trap onto the analytical column. Mobile phases were: A - 0.1% formic acid in water, and B - 0.1% formic acid in acetonitrile. The flow rate was programmed at 0.3 % to 35% 220 min. Mobile phase B was then increased from 35% to 80% in 5 min before being brought back quickly to 3% in 1 min. The column was equilibrated at 3% of mobile phase B for 15 min before the next sample. Peptides were eluted directly (300 nl min^{-1}) via a Triversa Nanomate nanospray source (Advion Biosciences, NY) into the Orbitrap mass spectrometer. Survey scans of peptide precursors from 350 to 1500 *m/z* were performed at 120K resolution (at 200 *m/z)* with automatic gain control (AGC) 4×10^5. Precursor ions with charge state 2 - 7 were isolated in 1.6 Th intervals in the quadrupole and subjected to high energy collision dissociation fragmentation programmed to 35% and fragments ions detected by rapid scan MS analysis in the ion trap; the AGC was set to 1×10^4 and the max injection time was 200 ms. Dynamic exclusion duration was set to 45 s with a 10 ppm tolerance around the selected precursor and its isotopes. Monoisotopic precursor selection was turned on. The instrument was run in top

speed mode with 2 s cycles. Sequence information from the MS/MS data was managed using MSConvert in ProteoWizard Toolkit (version 3.0.5759) [1] and searched with Mascot engine (Matrix Science, version 2.5.0) against *Homo sapiens* protein sequence database (http://www.uniprot.org/) assuming enzyme tryptic digestion to determine levels of false-positive peptide identifications; spectra were also searched against the corresponding reverse database, the common Repository of Adventitious Proteins Database (http://www.thegpm.org/cRAP/index.html). Search parameters for Precursor mass and product ions tolerance were, respectively, ± 5 ppm and ± 0.8 Da, with allowance made for two missed trypsin cleavages, fixed modification of cysteine through carbamidomethylation and methionine oxidation. Only fully tryptic peptide matches were allowed. Scaffold (version Scaffold 4.3.2, Proteome Software Inc.) was used to validate MS/MS based peptide and protein identifications from MS/MS sequencing results. Peptide identifications were accepted if they could be established at greater than 95.0% probability by the Scaffold Local FDR algorithm and contained at least 2 identified unique peptides; probabilities assigned by the Protein Prophet algorithm [2]. A relative quantification of protein concentration was performed using unique peptides in Progenesis see http://www.nonlinear.com/progenesis/qi-for-proteomics/v2.0/faq/which-quantitation-method-should-i-choose-for-my-experiment.aspx#relative-all for further details).

Protein ontology was evaluated using the Database for Annotation, Visualization and Integrated Discovery (DAVID) v6.8 (https://david.ncifcrf.gov/) [3] to identify molecular functions and biologic processes that may be impacted by changes in protein abundance and MG modification. REACTOME database was used (https://reactome.org/) for pathways analysis and INTERPRO analysis was performed (https://www.ebi.ac.uk/interpro/) for enrichment of protein domains modified by MG. Pathway and domain enrichment was

considered significant with Bonferroni significant and false positive discovery rate (FDR) of <0.05.

RBD analysis was performed using peptide window width of 5 residues and alpha-helix angle 100°, as found optimum by Gallet *at al.* [4]. It was applied to the 220 proteins detected with MG modification in the cytosolic protein extract of HMEC-1 cells (Table 4). Protein sequences were obtained from the UniProtKB/Swiss-Prot database. Non-standard amino acid codes in sequences were treated as follows: U (selenocysteine) as cysteine; B (asparagine or aspartic acid) as asparagine; Z (glutamine or glutamic acid) as glutamic acid; J (leucine or isoleucine) as leucine; and X (unknown or unspecified) as glycine. For each amino acid, mean alpha-helical hydrophobic moment (μ_H) and mean hydrophobicity (H) were calculated and plotted. The RBD area was defined as the trapezium bounded by $H<0.5$, $\mu_H>0.25$ and the equation $\mu_H = -0.4H + 0.6$. Arginine and MG-H1 residues which lay within the RBD were identified.

References

1 Kessner, D., Chambers, M., Burke, R., Agus, D. & Mallick, P. ProteoWizard: open source software for rapid proteomics tools development. *Bioinformatics* **24**, 2534-2536 (2008).

2 Nesvizhskii, A. I., Keller, A., Kolker, E. & Aebersold, R. A Statistical Model for Identifying Proteins by Tandem Mass Spectrometry. *Anal. Chem.* **75**, 4646-4658 (2003).

3 Huang, D. W., Sherman, B. T. & Lempicki, R. A. Systematic and integrative analysis of large gene lists using DAVID bioinformatics resources. *Nature Protocols* **4**, 44 (2008).

4 Gallet, X., Charloteaux, B., Thomas, A. & Braseur, R. A fast method to predict protein interaction sites from sequences. *J Mol Biol* **302**, 917-926 (2000).

Table S1. 231 Proteins increased in high glucose culture.

No	Protein (gene)	Fold increase	UNIPROT ID
1	Leukotriene A4 hydrolase (LTA4H)	3.54	LKHA4_HUMAN
2	Gamma-glutamylcyclotransferase (GGCT)	3.23	GGCT_HUMAN
3	5-Methyltetrahydrofolate-homocysteine methyltransferase (MTR)	3.16	METH_HUMAN
4	Glutamic-oxaloacetic transaminase 1 (GOT1)	2.93	AATC_HUMAN
5	G patch domain and KOW motifs-containing protein (GPKOW)	2.61	GPKOW_HUMAN
6	Thioredoxin reductase 1 (TXNRD1)	2.43	TRXR1_HUMAN
7	Actinin alpha 1 (ACTN1)	2.31	ACTN1_HUMAN
8	Glyceraldehyde-3-phosphate dehydrogenase (GAPDH)	2.23	G3P_HUMAN
9	Serpin family E member 1 (SERPINE1)	2.18	PAI1_HUMAN
10	SH3 domain-binding glutamic acid-rich-like protein (SH3BGRL)	2.11	SH3L1_HUMAN
11	Protein O-GlcNAcase (MGEA5)	2.09	OGA_HUMAN
12	Septin 10 (SEPT10)	2.09	SEP10_HUMAN
13	Transgelin (TAGLN)	2.07	TAGL_HUMAN
14	Myosin light chain 12A (MYL12A)	2.06	ML12A_HUMAN
15	Phosphatidylinositol transfer protein alpha (PITPNA)	2.05	PIPNA_HUMAN
16	Intersectin 1 (ITSN1)	1.99	ITSN1_HUMAN
17	Iron-responsive element-binding protein 2 (IREB2)	1.97	IREB2_HUMAN
18	Heme-binding protein 1 (HEBP1)	1.97	HEBP1_HUMAN
19	COP9 signalosome subunit 3 (COPS3)	1.88	CSN3_HUMAN
20	Signal peptidase complex subunit 2 (SPCS2)	1.87	SPCS2_HUMAN
21	CCR4-NOT transcription complex subunit 3 (CNOT3)	1.86	CNOT3_HUMAN
22	Nuclear migration protein (NUDC)	1.86	NUDC_HUMAN
23	Branched chain amino acid transaminase 1(BCAT1)	1.86	BCAT1_HUMAN
24	Twinfilin-2 (TWF2)	1.83	TWF2_HUMAN
25	N-Acetylglucosamine kinase (NAGK)	1.80	NAGK_HUMAN
26	Calponin 3 (CNN3)	1.80	CNN3_HUMAN
27	Cingulin like 1 (CGNL1)	1.78	CGNL1_HUMAN
28	Phosphoglycerate kinase 1 (PGK1)	1.78	PGK1_HUMAN
29	N-Myristoyltransferase 1 (NMT1)	1.76	NMT1_HUMAN
30	Minichromosome maintenance complex binding protein (MCMBP)	1.76	MCMBP_HUMAN
31	Negative regulator of ubiquitin like proteins 1 (NUB1)	1.74	NUB1_HUMAN
32	Methionine aminopeptidase 1 (METAP1)	1.71	MAP11_HUMAN
33	AP-3 complex subunit mu-1 (AP3M1)	1.70	AP3M1_HUMAN
34	A-kinase anchoring protein 13 (AKAP13)	1.70	AKP13_HUMAN
35	Cell division cycle 2, G1 to S and G2 to M, isoform CRA_a (CDC2)	1.69	CDK1_HUMAN
36	G-rich RNA sequence binding factor 1 (GRSF1)	1.68	GRSF1_HUMAN
37	Proteasome 26S subunit, non-ATPase 13 (PSMD13)	1.67	PSD13_HUMAN
38	Fructosamine 3 kinase related protein (FN3KRP)	1.67	KT3K_HUMAN
39	Leucine-rich repeat flightless-interacting protein 1 (LRRFIP1)	1.67	LRRF1_HUMAN
40	Phosphatidylethanolamine-binding protein 1 (PEBP1)	1.67	PEBP1_HUMAN
41	EH domain containing 1(EHD1)	1.67	EHD1_HUMAN
42	Parvin beta (PARVB)	1.66	PARVB_HUMAN
43	Activator of Hsp90 ATPase activity 1 (AHSA1)	1.66	AHSA1_HUMAN
44	Immunity-related GTPase family Q protein (IRGQ)	1.65	IRGQ_HUMAN
45	Serine/threonine-protein phosphatase 2A catalytic subunit alpha isoform (PPP2CA)	1.65	PP2AA_HUMAN
46	Fructose-bisphosphate aldolase C (ALDOC)	1.64	ALDOC_HUMAN
47	Glucosamine-6-phosphate deaminase 1 (GNPDA1)	1.64	GNPI1_HUMAN
48	Activating signal cointegrator 1 complex subunit 3 (ASCC3)	1.64	ASCC3_HUMAN
49	Set1/Ash2 histone methyltransferase complex subunit ASH2 (ASH2L)	1.63	ASH2L_HUMAN
50	Stress-70 protein, mitochondrial (HSPA9)	1.62	GRP75_HUMAN
51	Glutamate-cysteine ligase catalytic subunit (GCLC)	1.62	GSH1_HUMAN
52	Synaptopodin (SYNPO)	1.62	SYNPO_HUMAN
53	COP9 signalosome subunit 8 (COPS8)	1.62	CSN8_HUMAN
54	Tetratricopeptide repeat protein 1 (TTC1)	1.61	TTC1_HUMAN
55	Parvin alpha (PARVA)	1.61	PARVA_HUMAN
56	Importin 4 (IPO4)	1.60	IPO4_HUMAN
57	Splicing factor U2AF 65 kDa subunit (U2AF2)	1.60	U2AF2_HUMAN
58	Ubiquitin conjugating enzyme E2 V2 (UBE2V2)	1.60	UB2V2_HUMAN
59	Alcohol dehydrogenase [NADP(+)] (AKR1A1)	1.60	AK1A1_HUMAN
60	40S ribosomal protein S26 (RPS26)	1.60	RS26_HUMAN
61	Hedgehog interacting protein (HHIP)	1.59	HHIP_HUMAN
62	Cullin-5 (CUL5)	1.58	CUL5_HUMAN
63	Serine/arginine repetitive matrix 2 (SRRM2)	1.58	SRRM2_HUMAN

64	26S proteasome non-ATPase regulatory subunit 2 (PSMD2)	1.58	PSMD2_HUMAN
65	F-actin-capping protein subunit alpha-2 (CAPZA2)	1.58	CAZA2_HUMAN
66	Phosphoglucomutase 3 (PGM3)	1.58	AGM1_HUMAN
67	Elongation factor 1-beta (EEF1B2)	1.57	EF1B_HUMAN
68	Phosphoribosyl pyrophosphate synthetase 1 (PRPS1)	1.57	PRPS1_HUMAN
69	Serine/threonine-protein phosphatase 2A catalytic subunit beta isoform (PPP2CB)	1.57	PP2AB_HUMAN
70	Malate dehydrogenase 2 (MDH2)	1.56	MDHM_HUMAN
71	Peptidylprolyl isomerase H (PPIH)	1.56	PPIH_HUMAN
72	Glyoxalase domain containing 4 (GLOD4)	1.56	GLOD4_HUMAN
73	S-Methylthioadenosine phosphorylase (MTAP)	1.56	MTAP_HUMAN
74	Eukaryotic peptide chain release factor subunit 1 (ETF1)	1.55	ERF1_HUMAN
75	Glyoxylate reductase/hydroxypyruvate reductase (GRHPR)	1.55	GRHPR_HUMAN
76	Cleavage and polyadenylation specific factor 6 (CPSF6)	1.54	CPSF6_HUMAN
77	Tyrosine-protein phosphatase non-receptor type 23 (PTPN23)	1.54	PTN23_HUMAN
78	Prosaposin (PSAP)	1.54	SAP_HUMAN
79	Triosephosphate isomerase 1 (TPI1)	1.53	TPIS_HUMAN
80	Procollagen-lysine,2-oxoglutarate 5-dioxygenase 1(PLOD1)	1.52	PLOD1_HUMAN
81	N6-adenosine-methyltransferase 70 kDa subunit (METTL3)	1.52	MTA70_HUMAN
82	Protein phosphatase 6 regulatory subunit 3 (PPP6R3)	1.51	PP6R3_HUMAN
83	Ubiquitin specific peptidase 4 (USP4)	1.51	UBP4_HUMAN
84	Reticulocalbin 2 (RCN2)	1.51	RCN2_HUMAN
85	Thioredoxin-like protein 1 (TXNL1)	1.51	TXNL1_HUMAN
86	Epsin-1 (EPN1)	1.51	EPN1_HUMAN
87	Filamin B (FLNB)	1.50	FLNB_HUMAN
88	Prolyl 4-hydroxylase subunit alpha 2 (P4HA2)	1.50	P4HA2_HUMAN
89	Uncharacterized protein	1.50	A0A096LPI6
90	Carboxymethylenebutenolidase homolog (CMBL)	1.50	CMBL_HUMAN
91	Interferon-induced guanylate-binding protein 1 (GBP1)	1.50	GBP1_HUMAN
92	Translationally-controlled tumor protein1 (TPT1)	1.50	TCTP_HUMAN
93	Exportin 4 (XPO4)	1.49	XPO4_HUMAN
94	Pyruvate kinase, muscle (PKM)	1.49	KPYM_HUMAN
95	Ribosomal protein S24 (RPS24)	1.48	RS24_HUMAN
96	COP9 signalosome complex subunit 6 (COPS6)	1.48	CSN6_HUMAN
97	E2/E3 hybrid ubiquitin-protein ligase UBE2O (UBE2O)	1.47	UBE2O_HUMAN
98	Zinc finger CCHC domain-containing protein 8 (ZccHC8)	1.47	ZCHC8_HUMAN
99	Spectrin alpha, non-erythrocytic 1 (SPTAN1)	1.47	SPTN1_HUMAN
100	Tumor susceptibility 101 (TSG101)	1.46	TS101_HUMAN
101	Ornithine aminotransferase (OAT)	1.46	OAT_HUMAN
102	Thrombospondin 1 (THBS1)	1.46	TSP1_HUMAN
103	Cartilage associated protein (CRTAP)	1.46	CRTAP_HUMAN
104	Exportin-T (XPOT)	1.46	XPOT_HUMAN
105	BAG family molecular chaperone regulator 5 (BAG5)	1.46	BAG5_HUMAN
106	Supervillin (SVIL)	1.46	SVIL_HUMAN
107	UDP-glucose pyrophosphorylase 2(UGP2)	1.45	UGPA_HUMAN
108	ERO1-like protein alpha (ERO1L)	1.45	ERO1A_HUMAN
109	Peroxiredoxin 3 (PRDX3)	1.45	PRDX3_HUMAN
110	Probable cytosolic iron-sulfur protein assembly protein CIAO1 (CIAO1)	1.44	CIAO1_HUMAN
111	Heat shock cognate 71 kDa protein (HSPA8)	1.44	HSP7C_HUMAN
112	ATP-dependent DNA helicase Q1 (RECQL)	1.44	RECQ1_HUMAN
113	Dihydropyrimidinase like 3 (DPYSL3)	1.43	DPYL3_HUMAN
114	Proliferating cell nuclear antigen (PCNA)	1.42	PCNA_HUMAN
115	Procollagen-lysine,2-oxoglutarate 5-dioxygenase 3 (PLOD3)	1.41	PLOD3_HUMAN
116	Serine/threonine kinase 24 (STK24)	1.41	STK24_HUMAN
117	Transitional endoplasmic reticulum ATPase (VCP)	1.41	TERA_HUMAN
118	Phosphoglucomutase 2 (PGM2)	1.41	PGM2_HUMAN
119	Proteasome activator subunit 3 (PSME3)	1.40	PSME3_HUMAN
120	Filamin C (FLNC)	1.40	FLNC_HUMAN
121	Glutamine--tRNA ligase (QARS)	1.40	SYQ_HUMAN
122	Hexokinase 2 (HK2)	1.40	HXK2_HUMAN
123	Protein FAM49A (FAM49A)	1.40	FA49A_HUMAN
124	Calcineurin like phosphoesterase domain containing 1 (CPPED1)	1.39	CPPED_HUMAN
125	Nicotinamide N-methyltransferase (NNMT)	1.39	NNMT_HUMAN
126	Serine/threonine-protein kinase MRCK beta (CDC42BPB)	1.39	MRCKB_HUMAN
127	Eukaryotic translation elongation factor 1 epsilon 1(EEF1E1)	1.39	MCA3_HUMAN
128	PDZ and LIM domain 5 (PDLIM5)	1.39	PDLI5_HUMAN

129	Reticulocalbin-3 (RCN3)	1.39	RCN3_HUMAN
130	Actin-related protein 3 (ACTR3)	1.39	ARP3_HUMAN
131	Drebrin like (DBNL)	1.39	DBNL_HUMAN
132	Transportin 3 (TNPO3)	1.39	TNPO3_HUMAN
133	Filamin A (FLNA)	1.38	FLNA_HUMAN
134	tRNA-splicing ligase RtcB homolog (RTCB)	1.38	RTCB_HUMAN
135	Ubiquitin like modifier activating enzyme 6 (UBA6)	1.38	UBA6_HUMAN
136	Nucleoporin 214 (NUP214)	1.38	NU214_HUMAN
137	NudC domain containing 1 (NUDCD1)	1.38	NUDC1_HUMAN
138	Ras GTPase-activating-like protein (IQGAP1)	1.37	IQGA1_HUMAN
139	Replication protein A 70 kDa DNA-binding subunit (RPA1)	1.37	RFA1_HUMAN
140	Acetyl-CoA carboxylase beta (ACACB)	1.37	ACACB_HUMAN
141	Actin related protein 2/3 complex subunit 5 (ARPC5)	1.37	ARPC5_HUMAN
142	Cullin 2 (CUL2)	1.37	CUL2_HUMAN
143	Histidine-tRNA ligase, cytoplasmic (HARS)	1.37	SYHC_HUMAN
144	Phosphoglycerate mutase 1 (PGAM1)	1.37	PGAM1_HUMAN
145	Aminoacyl tRNA synthetase complex interacting multifunctional protein 1 (AIMP1)	1.36	AIMP1_HUMAN
146	Dihydropyrimidinase like 2 (DPYSL2)	1.36	DPYL2_HUMAN
147	WD repeat domain 61 (WDR61)	1.36	WDR61_HUMAN
148	Nuclear cap-binding protein subunit 1 (NCBP1)	1.36	NCBP1_HUMAN
149	Small nuclear ribonucleoprotein U5 subunit 200 (SNRNP200)	1.36	U520_HUMAN
150	Inorganic pyrophosphatase (PPA1)	1.36	IPYR_HUMAN
151	Endoplasmic reticulum aminopeptidase 1 (ERAP1)	1.36	ERAP1_HUMAN
152	Pre-mRNA-processing factor 19 (PRPF19)	1.36	PRP19_HUMAN
153	Fructose-2,6-bisphosphatase (TIGAR)	1.35	TIGAR_HUMAN
154	Gelsolin (GSN)	1.35	GELS_HUMAN
155	Nuclear pore complex protein Nup133 (NUP133)	1.34	NU133_HUMAN
156	ADP-ribosylation factor 1 (ARF1)	1.34	ARF1_HUMAN
157	RelA-associated inhibitor (PPP1R13L)	1.34	IASPP_HUMAN
158	Multifunctional protein ADE2 (PAICS)	1.34	PUR6_HUMAN
159	Calcyclin binding protein (CACYBP)	1.34	CYBP_HUMAN
160	SPARC (SPARC)	1.34	SPRC_HUMAN
161	Heat shock 70 kDa protein 1-like (HSPA1L)	1.34	HS71L_HUMAN
162	Heat shock protein beta-1 (HSPB1)	1.34	HSPB1_HUMAN
163	MCTS1, re-initiation and release factor (MCTS1)	1.34	MCTS1_HUMAN
164	Phosphomannomutase 2 (PMM2)	1.34	PMM2_HUMAN
165	E3 ubiquitin-protein ligase HECTD1 (HECTD1)	1.34	HECD1_HUMAN
166	Drebrin (DBN1)	1.34	DREB_HUMAN
167	Eukaryotic translation initiation factor 2 subunit 1 (EIF2S1)	1.33	IF2A_HUMAN
168	Proteasome 26S subunit, ATPase 3 (PSMC3)	1.33	PRS6A_HUMAN
169	Eukaryotic translation elongation factor 1 gamma (EEF1G)	1.33	EF1G_HUMAN
170	Acyl-CoA thioesterase 7 (ACOT7)	1.33	BACH_HUMAN
171	Laminin subunit beta 1(LAMB1)	1.33	LAMB1_HUMAN
172	Glucosidase 2 subunit beta (PRKCSH)	1.33	GLU2B_HUMAN
173	2-Aminoethanethiol dioxygenase (ADO)	1.33	AEDO_HUMAN
174	Importin subunit alpha-4 (KPNA3)	1.33	IMA4_HUMAN
175	Ran-specific GTPase-activating protein (RANBP1)	1.33	RANG_HUMAN
176	Adenylyl cyclase-associated protein 1 (CAP1)	1.32	CAP1_HUMAN
177	DNA mismatch repair protein (MSH2)	1.32	MSH2_HUMAN
178	Aldolase, fructose-bisphosphate A (ALDOA)	1.32	ALDOA_HUMAN
179	Heat shock 70 kDa protein 1A	1.32	HS71A_HUMAN
180	Heat shock 70 kDa protein 1B	1.32	HS71B_HUMAN
181	Ubiquitin-conjugating enzyme E2 N (UBE2N)	1.32	UBE2N_HUMAN
182	UDP-N-acetylglucosamine pyrophosphorylase 1 (UAP1)	1.32	UAP1_HUMAN
183	Phosphate cytidylyltransferase 1, choline, alpha (PCYT1A)	1.32	PCY1A_HUMAN
184	Peroxiredoxin 2 (PRDX2)	1.32	PRDX2_HUMAN
185	Protein flightless-1 homolog (FLII)	1.31	FLII_HUMAN
186	Catenin alpha 1 (CTNNA1)	1.31	CTNA1_HUMAN
187	RAB GTPase activating protein 1 (RABGAP1)	1.31	RBGP1_HUMAN
188	RuvB like AAA ATPase 2 (RUVBL2)	1.31	RUVB2_HUMAN
189	Calumenin (CALU)	1.31	CALU_HUMAN
190	SUMO-activating enzyme subunit 2 (UBA2)	1.31	SAE2_HUMAN
191	Signal recognition particle 54 kDa protein (SRP54)	1.31	SRP54_HUMAN
192	Procollagen-lysine,2-oxoglutarate 5-dioxygenase 2 (PLOD2)	1.30	PLOD2_HUMAN
193	Ribosomal protein S13 (RPS13)	1.30	RS13_HUMAN

194	Tubulin beta-4A chain (TUBB4A)	1.30	TBB4A_HUMAN
195	5-Aminoimidazole-4-carboxamide ribonucleotide formyltransferase/IMP cyclohydrolase(ATIC)	1.30	PUR9_HUMAN
196	Heat shock protein 105 kDa (HSPH1)	1.29	HS105_HUMAN
197	Chromobox protein homolog 3 (CBX3)	1.29	CBX3_HUMAN
198	Chloride intracellular channel protein 4 (CLIC4)	1.29	CLIC4_HUMAN
199	A-kinase anchoring protein 12 (AKAP12)	1.29	AKA12_HUMAN
200	Ras-related protein Rab-32 (RAB32)	1.29	RAB32_HUMAN
201	Diaphanous related formin 1 (DIAPH1)	1.29	DIAP1_HUMAN
202	WD repeat domain 1 (WDR1)	1.29	WDR1_HUMAN
203	Chaperonin containing TCP1 subunit 7 (CCT7)	1.29	TCPH_HUMAN
204	Actin, cytoplasmic 1 (ACTB)	1.29	ACTB_HUMAN
205	Heterogeneous nuclear ribonucleoprotein R (HNRNPR)	1.29	HNRPR_HUMAN
206	Calponin 2 (CNN2)	1.29	CNN2_HUMAN
207	EH domain-containing protein 4 (EHD4)	1.29	EHD4_HUMAN
208	Stromal cell derived factor 4 (SDF4)	1.29	CAB45_HUMAN
209	Adenylate kinase 1 (AK1)	1.29	KAD1_HUMAN
210	Talin-1 (TLN1)	1.29	TLN1_HUMAN
211	Nuclear mitotic apparatus protein 1 (NUMA1)	1.29	NUMA1_HUMAN
212	Importin-9 (IPO9)	1.29	IPO9_HUMAN
213	Ran GTPase-activating protein 1 (RANGAP1)	1.29	RAGP1_HUMAN
214	Splicing factor 1 (SF1)	1.29	SF01_HUMAN
215	Peptidyl-prolyl cis-trans isomerase (FKBP4)	1.29	FKBP4_HUMAN
216	E3 SUMO-protein ligase RanBP2 (RANBP2)	1.28	RBP2_HUMAN
217	Exportin-1 (XPO1)	1.28	XPO1_HUMAN
218	Cathepsin B (CTSB)	1.28	CATB_HUMAN
219	Protein disulfide isomerase family A member 3 (PDIA3)	1.28	PDIA3_HUMAN
220	SUMO1 activating enzyme subunit 1 (SAE1)	1.28	SAE1_HUMAN
221	SEC23-interacting protein (SEC23IP)	1.27	S23IP_HUMAN
222	Ribosome maturation protein SBDS (SBDS)	1.27	SBDS_HUMAN
223	Adaptor related protein complex 3 beta 1 subunit (AP3B1)	1.27	AP3B1_HUMAN
224	FK506 binding protein 15(FKBP15)	1.27	FKB15_HUMAN
225	Transducin beta like 3(TBL3)	1.26	TBL3_HUMAN
226	Glutaredoxin-3 (GLRX3)	1.26	GLRX3_HUMAN
227	Heterogeneous nuclear ribonucleoprotein C (C1/C2)(HNRNPC)	1.26	HNRPC_HUMAN
228	Methionyl-tRNA synthetase (MARS)	1.26	SYMC_HUMAN
229	Methionine adenosyltransferase 2A (MAT2A)	1.26	METK2_HUMAN
230	Diaphanous related formin 2 (DIAPH2)	1.26	Q17R91_HUMAN
231	78 kDa glucose-regulated protein (HSPA5)	1.26	GRP78_HUMAN
232	T-complex protein 1 subunit zeta (CCT6A)	1.26	TCPZ_HUMAN
233	Lamin-B1 (LNB1)	1.25	LMNB1_HUMAN
234	Scaffold attachment factor B2	1.25	SAFB2_HUMAN
235	Importin subunit alpha-5 (KPNA1)	1.25	IMA5_HUMAN
236	Thioredoxin domain containing 5 (TXNDC5)	1.25	TXND5_HUMAN
237	Reticulocalbin 1 (RCN1)	1.25	RCN1_HUMAN
238	Cysteinyl-tRNA synthetase (CARS)	1.25	SYCC_HUMAN
239	Tropomodulin-3 (TMOD3)	1.25	TMOD3_HUMAN
240	Histone-binding protein RBBP7 (RBBP7)	1.25	RBBP7_HUMAN
241	Fascin (FSCN1)	1.25	FSCN1_HUMAN
242	Ras suppressor protein 1 (RSU1)	1.24	RSU1_HUMAN
243	Glutamate-cysteine ligase regulatory subunit (GCLM)	1.24	GSH0_HUMAN
244	Clathrin light chain A (CLTA)	1.24	CLCA_HUMAN
245	Arginyl-tRNA synthetase (RARS)	1.24	SYRC_HUMAN
246	T-complex protein 1 subunit epsilon (CCT5)	1.24	TCPE_HUMAN
247	Polypyrimidine tract binding protein 1 (PTBP1)	1.24	PTBP1_HUMAN
248	Aldo-keto reductase family 1 member C3 (AKR1C3)	1.24	AK1C3_HUMAN
249	Proteasome 26S subunit, non-ATPase 12 (PSMD12)	1.24	PSD12_HUMAN
250	Adenylosuccinate synthetase isozyme 2 (ADSS)	1.24	PURA2_HUMAN
251	SON DNA binding protein (SON)	1.24	SON_HUMAN
252	Serine/threonine-protein kinase PRP4 homolog (PRPF4B)	1.24	PRP4B_HUMAN
253	Tubulin beta chain (TUBB)	1.24	TBB5_HUMAN
254	Component of oligomeric golgi complex 1 (COG1)	1.24	COG1_HUMAN
255	Lysyl-tRNA synthetase (KARS)	1.23	SYK_HUMAN
256	Histone deacetylase 2(HDAC2)	1.23	HDAC2_HUMAN
257	Cytochrome c, somatic (CYCS)	1.23	CYC_HUMAN
258	Synaptotagmin binding cytoplasmic RNA interacting protein (SYNCRIP)	1.23	HNRPQ_HUMAN

259	Ubiquitin specific peptidase 39 (USP39)	1.23	SNUT2_HUMAN
260	Histone deacetylase 1 (HDAC1)	1.23	HDAC1_HUMAN
261	Peroxiredoxin-1 (PRDX1)	1.23	PRDX1_HUMAN
262	Sulfotransferase family cytosolic 1B member 1 (SULT1B1)	1.23	ST1B1_HUMAN
263	Aspartyl-tRNA synthetase (DARS)	1.22	SYDC_HUMAN
264	Tubulin beta-4B chain (TUBB4B)	1.22	TBB4B_HUMAN
265	Calpastatin (CAST)	1.22	ICAL_HUMAN
266	Adaptor related protein complex 3 delta 1 subunit (AP3D1)	1.22	AP3D1_HUMAN
267	Vinculin (VCL)	1.22	VINC_HUMAN
268	Tripartite motif containing 28 (TRIM28)	1.21	TIF1B_HUMAN
269	Utrophin (UTRN)	1.21	UTRO_HUMAN
270	Ubiquitin protein ligase E3C (UBE3C)	1.21	UBE3C_HUMAN
271	C-Jun-amino-terminal kinase-interacting protein 4 (SPAG9)	1.21	JIP4_HUMAN
272	Microtubule-actin cross-linking factor 1, isoforms 1/2/3/5 (MACF1)	1.21	E9PLY5_HUMAN
273	Hypoxia up-regulated 1 (HYOU1)	1.21	HYOU1_HUMAN
274	Serum deprivation-response protein (SDPR)	1.20	SDPR_HUMAN
275	Carbamoyl-phosphate synthetase 2, aspartate transcarbamylase, and dihydroorotase (CAD)	1.20	PYR1_HUMAN
276	26S proteasome non-ATPase regulatory subunit 1 (PSMD1)	1.20	PSMD1_HUMAN
277	CAP-Gly domain containing linker protein 1(CLIP1)	1.20	CLIP1_HUMAN
278	14-3-3 protein gamma (1433G)	1.20	1433G_HUMAN
279	Phosphofructokinase, platelet (PFKP)	1.20	PFKAP_HUMAN
280	Rho-associated protein kinase 1 (ROCK1)	1.20	ROCK1_HUMAN
281	Protein phosphatase 1 regulatory subunit 14B (PPP1R14B)	1.20	PP14B_HUMAN
282	Protein S100-A13 (S100A13)	1.20	S10AD_HUMAN
283	Adaptor protein (CRK)	1.19	CRK_HUMAN
284	Trifunctional purine biosynthetic protein adenosine-3 (GART)	1.19	PUR2_HUMAN
285	40S ribosomal protein S17-like (RPS17L)	1.19	RS17_HUMAN
286	Kinesin-1 heavy chain (KIF5B)	1.19	KINH_HUMAN
287	Rho-associated protein kinase 2 (ROCK2)	1.19	ROCK2_HUMAN
288	Autophagy related 7 (ATG7)	1.19	ATG7_HUMAN
289	Actin related protein 2/3 complex subunit 1A (ARPC1A)	1.19	ARC1A_HUMAN
290	Prolyl 4-hydroxylase subunit alpha 1 (P4HA1)	1.18	P4HA1_HUMAN
291	Probable rRNA-processing protein EBP2 (EBNA1BP2)	1.18	EBP2_HUMAN
292	Calreticulin (CALR)	1.18	CALR_HUMAN
293	Isoform 2 of BH3-interacting domain death agonist (BID)	1.18	BID_HUMAN
294	Leucyl-tRNA synthetase (LARS)	1.18	SYLC_HUMAN
295	Staphylococcal nuclease domain-containing protein 1 (SND1)	1.18	SND1_HUMAN
296	RAC-alpha serine/threonine-protein kinase (AKT1)	1.18	AKT1_HUMAN
297	Phosphoprotein enriched in astrocytes 15(PEA15)	1.18	PEA15_HUMAN
298	Proteasome 26S subunit, ATPase 6 (PSMC6)	1.18	PRS10_HUMAN
299	ATP-dependent RNA helicase A (DHX9)	1.17	DHX9_HUMAN
300	Nicotinate phosphoribosyltransferase (NAPRT)	1.17	PNCB_HUMAN
301	Microtubule-actin crosslinking factor 1 (MACF1)	1.17	MACF1_HUMAN
302	Myristoylated alanine-rich C-kinase substrate (MARCKS)	1.17	MARCS_HUMAN
303	GDP-mannose pyrophosphorylase B (GMPPB)	1.17	GMPPB_HUMAN
304	Ubiquitin like modifier activating enzyme 1 (UBA1)	1.16	UBA1_HUMAN
305	Kinesin family member 13B (KIF13B)	1.16	KI13B_HUMAN
306	Protein disulfide-isomerase A4 (PDIA4)	1.16	PDIA4_HUMAN
307	Ribosomal protein L32 (RPL32)	1.16	RL32_HUMAN
308	Dedicator of cytokinesis protein 6 (DOCK6)	1.16	DOCK6_HUMAN
309	Tight junction protein 1 (TJP1)	1.15	ZO1_HUMAN
310	Pre-rRNA processing protein FTSJ3 (FTSJ3)	1.15	SPB1_HUMAN
311	Basement membrane-specific heparan sulfate proteoglycan core protein (HSPG2)	1.15	PGBM_HUMAN
312	Protein phosphatase 1 regulatory subunit 12A (PPP1R12A)	1.15	MYPT1_HUMAN
313	Mago homolog, exon junction complex core component (MAGOH)	1.13	MGN_HUMAN
314	Family with sequence similarity 129 member B (FAM129B)	1.13	NIBL1_HUMAN
315	Leucine aminopeptidase 3 (LAP3)	1.13	AMPL_HUMAN
316	UPF0568 protein C14orf166 (C14orf166)	1.13	CN166_HUMAN
317	Bifunctional glutamate/proline-tRNA ligase (EPRS)	1.12	SYEP_HUMAN
318	NCK associated protein 1 (NCKAP1)	1.11	NCKP1_HUMAN
319	Proteasome 26S subunit, non-ATPase 3(PSMD3)	1.11	PSMD3_HUMAN
320	Transglutaminase 2 (TGM2)	1.11	TGM2_HUMAN
321	Eukaryotic translation initiation factor 5B (EIF5B)	1.11	IF2P_HUMAN
322	Dynein light chain 1, cytoplasmic (DYNLL1)	1.11	DYL1_HUMAN
323	Cytoplasmic dynein 1 heavy chain 1 (DYNC1H1)	1.11	DYHC1_HUMAN

324	Peptidyl-prolyl cis-trans isomerase (FKBP5)	1.10	FKBP5_HUMAN
325	Actin-related protein 2/3 complex subunit 1B (ARPC1B)	1.10	ARC1B_HUMAN
326	ATP-dependent RNA helicase (DDX1)	1.10	DDX1_HUMAN
327	Poly(rC)-binding protein 1 (PCBP1)	1.10	PCBP1_HUMAN
328	Unconventional myosin-Ie (MYO1E)	1.09	MYO1E_HUMAN
329	Dynamin 1 like (DNM1L)	1.09	DNM1L_HUMAN
330	interleukin enhancer binding factor 3 (ILF3)	1.08	ILF3_HUMAN
331	Hepatoma-derived growth factor (HDGF)	1.08	HDGF_HUMAN

Table S2. 49 Proteins decreased in high glucose culture.

No	Protein	Fraction of control	UNIPROT entry name
1	Copine-1 (CPNE1)	0.19	CPNE1_HUMAN
2	Annexin A1 (ANXA1)	0.19	ANXA1_HUMAN
3	Annexin A5 (ANXA5)	0.26	ANXA5_HUMAN
4	Adipogenesis regulatory factor (ADIRF)	0.40	ADIRF_HUMAN
5	Proline-rich and coiled-coil-containing protein 1 (PRRC1)	0.47	PRRC1_HUMAN
6	Zinc finger CCCH domain-containing protein 18 (ZC3H18)	0.52	ZCH18_HUMAN
7	RNA-binding protein 4 (RBM4)	0.52	RBM4_HUMAN
8	RNA-binding motif, single-stranded-interacting protein 1 (RBMS1)	0.52	RBMS1_HUMAN
9	Cell division cycle protein 16 homolog (CDC16)	0.54	CDC16_HUMAN
10	Putative helicase (MOV10)	0.56	MOV10_HUMAN
11	Endophilin-A2 (SH3GL1)	0.59	SH3G1_HUMAN
12	Ras-related protein Rab-7a	0.59	RAB7A_HUMAN
13	Nucleoporin 153 (NU153)	0.62	NU153_HUMAN
14	Triple functional domain protein (TRIO)	0.64	TRIO_HUMAN
15	Tetratricopeptide repeat protein 37 (TTC37)	0.64	TTC37_HUMAN
16	Syntaxin-12 (STX12)	0.64	STX12_HUMAN
17	Microfibrillar-associated protein 1 (MFAP1)	0.64	MFAP1_HUMAN
18	Eukaryotic translation initiation factor 4E (EIF4E)	0.65	IF4E_HUMAN
19	ATP-dependent RNA helicase (DDX54)	0.66	DDX54_HUMAN
20	Phosphatidylinositol-binding clathrin assembly protein (PICALM)	0.66	PICAL_HUMAN
21	Cytokine-induced apoptosis inhibitor 1 (CIAPIN1)	0.67	CPIN1_HUMAN
22	Muscle blind-like protein 1 (MBNL1)	0.68	MBNL1_HUMAN
23	Alpha-synuclein (SNCA)	0.70	SYUA_HUMAN
24	Cell growth-regulating nucleolar protein (LYAR)	0.70	LYAR_HUMAN
25	Chromobox protein homolog 5 (CBX5)	0.70	CBX5_HUMAN
26	60S ribosomal protein L35 (RPL35)	0.70	RL35_HUMAN
27	Putative phospholipase B-like 2 (PLBD2)	0.72	PLBL2_HUMAN
28	Adenylyl cyclase-associated protein 2 (CAP2)	0.72	CAP2_HUMAN
29	UBX domain-containing protein 1 (UBXN1)	0.73	UBXN1_HUMAN
30	Cohesin subunit SA-2 (STAG2)	0.73	STAG2_HUMAN
31	RNA-binding protein Nova-2 (NOVA2)	0.73	NOVA2_HUMAN
32	Pumilio homolog 3 (PUM3)	0.73	PUM3_HUMAN
33	40S ribosomal protein S20 (RPS20)	0.74	RS20_HUMAN
34	Bystin (BYSL)	0.74	BYST_HUMAN
35	40S ribosomal protein S2 (RPS2)	0.76	RS2_HUMAN
36	NHL repeat-containing protein 2 (NHLRC2)	0.77	NHLC2_HUMAN
37	X-ray repair cross-complementing protein 5 (XRCC5)	0.77	XRCC5_HUMAN
38	U3 small nucleolar RNA-associated protein 6 homolog (UTP6)	0.78	UTP6_HUMAN
39	Glyoxalase 1 (GLO1)	0.79	LGUL_HUMAN
40	Transcription elongation factor A protein 1 (TCEA1)	0.79	TCEA1_HUMAN
41	D-3-Phosphoglycerate dehydrogenase (PHGDH)	0.81	SERA_HUMAN
42	Nucleolar complex protein 4 homolog (NOC4L)	0.82	NOC4L_HUMAN
43	Striatin-interacting protein 1 (STRIP1)	0.83	STRP1_HUMAN
44	26S proteasome non-ATPase regulatory subunit 4 (PSMD4)	0.84	PSMD4_HUMAN
45	60S ribosomal protein L13a (RPL13A)	0.84	RL13A_HUMAN
46	Interferon-inducible double-stranded RNA-dependent protein kinase activator A (PRKRA)	0.85	PRKRA_HUMAN
47	60S ribosomal protein L5 (RPL5)	0.86	RL5_HUMAN
48	Lambda-crystallin homolog (CRYL1)	0.91	CRYL1_HUMAN
49	Proliferation-associated protein 2G4 (PA2G4)	0.93	PA2G4_HUMAN

Table S3 Dicarbonyl proteome in cytosolic extract with exogenous MG modification.

Protein no	Protein	Number of sites	Total arg	MG Modification sites	MG Modification sites in the RBD
1	Pyruvate kinase-M (PKM)	11	31	R43, R56, R73, R106, R120, R278, R376, R383, R400, R461, R455	R43
2	β-Actin (ACTB)	9	19	R37, R39, R62, R177, R183, R196, R206, R256, R312	R37, R39, R196, R256, R312
3	α-Enolase (ENO1)	9	17	R15, R32, R50, R56, R132, R179, R183, R269, R412	R50, R56
4	Heat shock protein HSP 90-beta (HSP90AB1)	9	32	R41, R82, R168, R197, R330, R338, R475, R504, R612	R82
5	Moesin (MOES)	7	40	R184, R233, R246, R427, R435, R460, R533	R233
6	Transketolase (TKT)	6	29	R246, R334, R474, R530, R609, R617	R474
7	Actin, γ-enteric smooth muscle (ACTG2)	6	18	R29, R38, R40, R63, R184, R257	R38, R40, R257
8	Glucose-6-phosphate isomerase (G6PI)	6	27	R66, R83, R96, R106, R370, R438	R83
9	Tubulin alpha-1A chain	6	21	R64, R79, R105, R121, R123, R221	R121, R123
10	Heat shock protein HSP 90-alpha (HSP90AA1)	6	30	R46, R87, R201, R202, R620, R647	R87, R201, R202, R620, R647
11	Aldehyde dehydrogenase 16A1 (A16A1)	5	58	R117, R322, R334, R567, R629	R322
12	Glyceraldehyde-3-phosphate dehydrogenase (G3P)	5	10	R80, R118, R200, R234, R248	R80
13	Tropomyosin α4 chain (TPM4)	5	17	R27, R55, R89, R97, R123	R27, R55, R123
14	14-3-3 protein beta (1433B)	4	10	R20, R43, R62, R129	R20, R43
15	14-3-3 protein zeta (1433Z)	4	11	R41, R56, R60, R127	R41, R56
16	Actin, cytoplasmic 2 (ACTG1)	4	13	R28, R37, R39, R62	R37, R39
17	Alpha-actin-1 (ACTA1)	4	10	R39, R41, R64, R97	R39, R41
18	Alpha-actin-2 (ACTA2)	4	16	R39, R41, R185, R258	R39, R41, R258
19	β-Actin-like protein 2 (ACTBL2)	4	20	R184, R197, R207, R257	R197, R257
20	Annexin A1 (ANXA1)	4	19	R124, R144, R177, R228	R124, R144, R228
21	Elongation factor 2 (EEF2)	4	44	R10, R20, R506, R726	R726
22	Heat shock cognate 71 kDa protein (HSPA8)	4	28	R77, R100, R301, R342	R77, R100
23	L-lactate dehydrogenase A chain (LDHA)	4	11	R99, R157, R169, R315	R99, R169
24	Phosphoglycerate kinase 1 (PGK1)	4	11	R39, R151, R171, R206	R39
25	Protein disulfide-isomerase A3 (PDIA3)	4	23	R37, R304, R326, R423	
26	T-complex protein 1 subunit beta (CCT2)	4	22	R57, R89, R138, R156	R57, R156
27	Thyroid autoantigen 70kDa (XRCC6)	4	32	R35, R194, R290, R301	R35, R194, R301
28	Tropomyosin α3 chain (TPM3)	4	18	R126, R134, R161, R168	R126, R161
29	14-3-3 protein gamma (1433G)	3	13	R12, R19, R61	R19
30	40S ribosomal protein S3 (RPS3)	3	18	R45, R67, R76	R45, R67, R76
31	78 kDa glucose-regulated protein (GRP78)	3	28	R97, R101, R181	R181
32	Aspartate aminotransferase, cytoplasmic (AATM)	3	21	R68, R337, R407	R337
33	Elongation factor 1-alpha 2 (EEF1A2)	3	18	R134, R427, R430	R134, R430
34	Ezrin (EZR)	3	46	R40, R151, R246	
35	Inosine-5'-monophosphate dehydrogenase 2 (IMPDH2)	3	22	R182, R412, R493	R182, R412, R493
36	L-lactate dehydrogenase B chain (LDHB)	3	8	R107, R170, R172	R107, R170, R172
37	Proteasome subunit B5 (PSMB5)	3	18	R68, R132, R166	R132, R166
38	Ribosomal protein L5 (RPL5)	3	24	R179, R209, R248	R179

39	Ribosomal protein L15 (RL15)	3	25	R96, R105, R108	R105
40	T-complex protein 1 subunit delta (TPCD)	3	28	R49, R206, R240	
41	Transitional endoplasmic reticulum ATPase (VCP)	3	58	R225, R287, R323	R287, R323
42	Triosephosphate isomerase (TPI1)	3	11	R137, R172, R243	R137, R172
43	Tubulin beta (TUBB)	3	22	R121, R241, R251	R121, R251
44	Tumor protein D54 (TPD52L2)	3	8	R153, R170, R182	R170, R182
45	14-3-3 protein epsilon (1433E)	2	14	R57, R61	R57
46	14-3-3 protein eta (1433F)	2	12	R42, R61	R42
47	40S ribosomal protein S7 (TPD54)	2	14	R41, R120	R120
48	40S ribosomal protein S8 (RPS8)	2	23	R77, R92	
49	40S ribosomal protein S19 (RPS19)	2	11	R16, R56	
50	60S ribosomal protein L7 (RPL7)	2	27	R154, R166	R166
51	60S ribosomal protein L23a (RPL23A)	2	10	R129, R139	
52	60S acidic ribosomal protein P0 (RPLP0)	2	14	R149, R247	
53	Aconitate hydratase, mitochondrial (ACO2)	2	35	R607, R632	R607
54	Annexin A5 (ANXA5)	2	18	R123, R245	
55	Alanine--tRNA ligase, cytoplasmic (AARS)	2	47	R320, R751	
56	Caveolae-associated protein 1 (CAVIN1)	2	29	R130, R329	R329
57	Far upstream element-binding protein 2 (KHSRP)	1	30	R331, R340	
58	Filamin A (FLNA)	2	93	R1060, R1782	
59	GTP-binding nuclear protein Ran (RAN)	2	9	R29, R106	
60	Heat shock protein 70 kDa 1A (HSP71A)	2	41	R342, R357	
61	Heterogeneous nuclear ribonucleoproteins C (HNRPC)	2	18	R17, R73	
62	Endoplasmin (HSP90B1)	2	16	R84, R102	
63	Nucleolin (NCL)	2	26	R597, R599	
64	Peroxiredoxin-1 (PRDX1)	2	6	R110, R128	R110
65	Peroxiredoxin-5 (PRDX5)	2	11	R148, R180	
66	Peroxiredoxin-6 (PRDX6)	2	12	R132, R174	R174
67	Proteasome subunit A3 (PSMA3)	2	13	R72, R233	R233
68	Proteasome subunit A7 (PSMA7)	2	18	R38, R213	R38, R213
69	Protein disulfide-isomerase A1 (PDIA1)	2	15	R106, R452	
70	Protein disulfide-isomerase A3 (PDIA3)	1	23	R38	
71	Protein disulfide-isomerase A4 (PDIA4)	2	23	R131, R155	R155
72	Rho GDP-dissociation inhibitor 2 (ARHGD1B)	2	6	R131	
73	Ribose-phosphate pyrophosphokinase 2 (PRPS2)	2	15	R177, R302	R302
74	Ribosomal protein L19 (RPL19)	2	31	R162, R163	R162, R163
75	Serine hydroxymethyltransferase (GLYC)	2	29	R272, R355	
76	SH3 domain-binding protein 5-like (SH3BP5L)	2	40	R19, R51	R19, R51
77	Stress-70 protein, mitochondrial (HSPA9)	2	35	R146, R405	
78	Stress-induced-phosphoprotein 1 (STIP1)	2	27	R355, R443	
79	T-complex protein 1 subunit theta (TPCQ)	2	18	R165, R407	R407
80	Transglutaminase-2 (TGM2)	2	38	R476, R680	
81	TubulinTubulin alpha-1C chain (TUBA1C)	2	19	R79, R105	
82	Vimentin (VIM)	2	43	R401, R223	R401
83	WD repeat-containing protein 1 (WDR1)	2	18	R116, R396	

84	10 kDa heat shock protein, mitochondrial (HSPE1)	1	4	R20	
85	40S Ribosomal protein S2 (RPS2)	1	24	R284	
86	40S ribosomal protein S3a (RPS3A)	1	14	R174	R284
87	40S ribosomal protein S4	1	22	R77	
88	40S ribosomal protein S6 (RPS6)	1	31	R31	R77
89	40S ribosomal protein S9 (RPS9)	1	16	R24	R31
90	40S ribosomal protein S10 (RPS10)	1	16	R8	
91	40S ribosomal protein S11 (RPS11)	1	14	R22	
92	40S ribosomal protein S18 (RPS18)	1	20	R86	R22
93	40S ribosomal protein S27 (RPS27)	1	2	R23	R86
94	60S acidic ribosomal protein P2 (RPLP2)	1	2	R38	R23
95	60S ribosomal protein L6 (RPL6)	1	23	R156	R38
96	60S ribosomal protein L10a (RPL10A)	1	11	R7	R156
97	60S ribosomal protein L12 (RPL12)	1	10	R117	R7
98	60S ribosomal protein L13 (RPL13)	1	31	R116	
99	60S ribosomal protein L13a (RPL13A)	1	22	R12	R116
100	60S ribosomal protein L22 (RPL22)	1	6	R65	R12
101	60S ribosomal protein L27 (RPL27)	1	13	R102	R65
102	60S Ribosomal protein L27a (RPL27A)	1	13	R105	
103	60S ribosomal protein L30 (RPL30)	1	5	R17	
104	60S ribosomal protein L31 (RPL31)	1	16	R14	R17
105	60S ribosomal protein L34 (RPL34)	1	14	R93	R14
106	116 kDa U5 small nuclear ribonucleoprotein component (EFTUD2)	1	41	R803	
107	Acidic leucine-rich nuclear phosphoprotein 32 (ANP32A)	1	11	R150	
108	Actin related protein 2/3 complex subunit 1A (ARPC1A)	1	17	R89	R150
109	Actin related protein 2/3 complex, 1B, 41kDa (ARPC1B)	1	17	R179	
110	Actin-related protein 2/3 complex subunit 2 (ARPC2)	1	17	R78	
111	Actin-related protein 2/3 complex subunit 4 (ARPC4)	1	12	R158	R179
112	Actin-related protein 2/3 complex subunit 5 (ARPC5)	1	6	R74	
113	Actin-related protein 3 (ACTR3)	1	24	R79	
114	Adenosylhomocysteinase (AHCY)	1	17	R205	R74
115	S-Adenosylmethionine synthase isoform type-2 (MAT2A)	1	20	R343	
116	ADP-ribose pyrophosphatase, mitochondrial (NUDT9)	1	21	R247	
117	Alpha-actinin-4 (ACTN4)	1	47	R869	R343
118	Annexin A2 (ANXA2)	1	21	R168	R247
119	Annexin A2-like protein (ANXA2P2)	1	20	R168	R869
120	Arginine--tRNA ligase (RARS)	1	35	R659	
121	Asparagine synthetase [glutamine-hydrolyzing] (ASNS)	1	27	R143	
122	Asparagine--tRNA ligase, cytoplasmic (NARS)	1	38	R390	
123	Aspartate-tRNA ligase, cytoplasmic (DARS)	1	34	R320	
124	Astrocytic phosphoprotein PEA-15 (PEA15)	1	6	R101	R390
125	ATP-citrate synthase (ACLY)	1	48	R607	R320
126	ATP-dependent 6-phosphofructokinase, platelet type (PFKP)	1	48	R44	
127	ATP-dependent RNA helicase A (DHX9)	1	73	R447	
128	Bifunctional glutamate/proline-tRNA ligase (EPRS)	1	56	R1463	

129	Calreticulin (CALR)	1	8	R222	
130	Casein kinase II subunit alpha (CSNK2A1)	1	27	R191	R1463
131	Casein kinase II subunit alpha 3 (CSNK2A3)	1	28	R191	R222
132	Cathepsin Z (CTSZ)	1	21	R199	
133	Coactosin-like protein (COTL1)	1	7	R91	R91
134	Cytosolic acyl coenzyme A thioester hydrolase (ACOT7)	1	28	R61	
135	Dihydropteridine reductase (QDPR)	1	9	R236	R236
136	Dihydropyrimidinase-related protein 2 (DPYSL2)	1	32	R268	R268
137	DNA damage-binding protein 1 (DDB1)	1	47	R1138	
138	DNA topoisomerase 2-alpha (TOP2A)	1	66	R804	
139	Electron transfer flavoprotein subunit α, mitochondrial (ETFA)	1	11	R169	
140	Elongin B (ELOB)	1	7	R24	
141	γ-Enolase (ENO2)	1	20	R32	
142	Eukaryotic translation initiation factor 3 subunit F	1	12	R108	
143	Fascin (FSCN1)	1	32	R208	
144	Filamin-B (FLNB)	1	81	R2578	
145	Filamin-C (FLNC)	1	110	R2293	
146	Fructose-bisphosphate aldolase A (ALDOA)	1	15	R43	R43
147	Fumarate hydratase, mitochondrial (FH)	1	19	R101	
148	Galectin-1 (LGALS1)	1	5	R21	
149	Glucose-6-phosphate dehydrogenase (G6PDH)	1	32	R458	
150	Glutamine--tRNA synthetase (QARS)	1	50	R576	
151	Glutathione S-transferase omega 1 (GSTO1)	1	9	R48	
152	Glycine-tRNA ligase (GARS)	1	43	R602	R602
153	Heat shock protein beta-1 (HSPB1)	1	16	R188	R188
154	Heterogeneous nuclear ribonucleoprotein A/B (HNRNPAB)	1	13	R195	
155	Heterogeneous nuclear ribonucleoproteins A2/B1 (HNRNPA2B1)	1	25	R153	R153
156	Heterogeneous nuclear ribonucleoprotein K (HNRPK)	1	36	R69	
157	Heterogeneous nuclear ribonucleoprotein Q (SYNCRIP)	1	45	R343	
158	Heterogeneous nuclear ribonucleoprotein R (HNRPR)	1	47	R308	R308
159	Heat shock 70 kDa protein 4 (HSPA4)	1	29	R234	
160	Insulin-like growth factor 2 mRNA-binding protein 2 (IF2B2)	1	29	R576	
161	Interleukin enhancer-binding factor 2 (ILF2)	1	23	R60	R60
162	Interleukin enhancer-binding factor 3 (ILF3)	1	38	R264	
163	Leucine-tRNA ligase, cytoplasmic (LARS)	1	49	R785	
164	Lysine-tRNA ligase (KARS)	1	28	R314	
165	Malate dehydrogenase, mitochondrial (MDH2)	1	10	R52	
166	S-Methyl-5'-thioadenosine phosphorylase (MTAP)	1	19	R166	
167	Microtubule-associated protein 4, similar (B4DM10_HUMAN)	1	10	R583	
168	Multifunctional protein ADE2 (PAICS)	1	16	R255	
169	Myosin-9 (MYH9)	1	122	R358	
170	Neutral alpha-glucosidase AB (GANAB)	1	66	R944	
171	Nicotinamide phosphoribosyltransferase (NAMPT)	1	14	R196	
172	Nicotinamide N-methyltransferase (NNMT)	1	9	R30	R30
173	Nucleosome assembly protein 1-like 1 (NAPIL1)	1	13	R104	R104

174	Nucleosome assembly protein 1-like 4 (NAPIL4)	1	14	R93	R93
175	Nucleoside diphosphate kinase (NME1-NME2)	1	16	R113	
176	Nuclear ubiquitous casein and cyclin-dependent kinase substrate 1	1	14	R28	R28
177	Peptidyl-prolyl cis-trans isomerase A (PPIA)	1	6	R37	
178	Phosphatase 2A Inhibitor (I2PP2A)	1	10	R136	
179	6-Phosphogluconate dehydrogenase, decarboxylating (PGD)	1	20	R382	R382
180	Phosphoglycerate mutase 1 (PGAM1)	1	17	R90	R90
181	Glycogen phosphorylase (PYGB)	1	53	R640	R640
182	Phosphoserine aminotransferase (PSAT1)	1	11	R45	R45
183	Plastin-3 (PLST)	1	25	R599	
184	Profilin-1 (PROF1)	1	5	R56	
185	Prostaglandin reductase 1 (PTGR1)	1	8	R56	R56
186	26S protease regulatory subunit 4 (PSMC1)	1	21	R249	
187	Proteasome subunit A2 (PSMA2)	1	10	R220	
188	Proteasome subunit A5 (PSMA5)	1	7	R53	R53
189	Proteasome subunit B2 (PSMB2)	1	12	R70	R70
190	Proteasome subunit B3 (PSMB3)	1	12	R27	
191	Proteasome subunit B4 (PSMB4)	1	18	R89	
192	Prothymosin alpha protein (PTMA)	1	2	R89	
193	Purine nucleoside phosphorylase (PNPH)	1	16	R101	
194	Isoleucine-tRNA ligase, cytoplasmic (IARS)	1	57	R624	
195	Ras-related protein Rab 1A (RAB1A)	1	7	R51	
196	Ras-related protein Rab 1B (RAB1B)	1	8	R48	
197	Receptor of activated protein C kinase 1 (RACK1)	1	13	R47	R47
198	Rho GDP-dissociation inhibitor 1 (ARHGD1A)	1	9	R49	
199	Serine/threonine-protein phosphatase PP1-alpha catalytic subunit (PPP1CA)	1	18	R43	
200	Serine/threonine-protein phosphatase PP1-beta catalytic subunit (PPP1CB)	1	21	R42	R42
201	Serine-tRNA ligase, cytoplasmic (SARS)	1	32	R107	
202	Sialic acid synthetase (NANS)	1	13	R271	
203	Spectrin beta non-erythrocytic 1 (SPTBN1)	1	152	R675	
204	Succinyl-CoA:3-ketoacid coenzyme A transferase 1, mitochondrial (OXCT1)	1	23	R104	
205	T-complex protein 1 subunit zeta (CCT6A)	1	25	R28	
206	T-complex protein 1 subunit alpha (TCP1)	1	25	R526	
207	T-complex protein 1 subunit epsilon (TPCE)	1	25	R49	
208	T-complex protein 1 subunit gamma (TPCG)	1	35	R518	
209	TIP41-like protein (TIPRL)	1	13	R200	
210	Transaldolase (TALDO)	1	14	R295	
211	Transforming protein RhoA (RHOA)	1	12	R168	
212	Tropomyosin 1alpha (TPM1)	1	17	R133	R133
213	Tubulin beta-4B chain (TUBB4B)	1	21	R251	
214	Tubulin beta-7 chain (B4DMJ5_HUMAN)	1	10	R121	
215	Ubiquitin carboxyl-terminal hydrolase (USP14)	1	16	R267	
216	Ubiquitin-like modifier-activating enzyme 1 (UBA1)	1	50	R557	
217	UDP-N-acetylhexosamine pyrophosphorylase (UAP1)	1	19	R399	
218	UTP-glucose-1-phosphate uridylyltransferase 2 (UGP2)	1	21	R301	
219	Vinculin (VINC)	1	67	R1107	

220	X-ray repair cross-complementing protein 5 (XRCC5)	1	26	R141	
TOTAL: 220		TOTAL 411			TOTAL:148 sites; 104 proteins

Table S4 REACTOME Pathways analysis of cytosolic proteins at risk of MG modification.

Reactome term	Process	Reactome pathway	Count	Fold enrichment	Bonferroni	FDR
R-HSA-390450:R-HSA-390450	Protein folding	Folding of actin by CCT/TriC	8	38.6	4.53E-08	2.13E-07
R-HSA-2408517:R-HSA-2408517	Protein synthesis	SeMet incorporation into proteins	7	30.7	8.45E-06	3.97E-05
R-HSA-379716:R-HSA-379716	Protein synthesis	Cytosolic tRNA aminoacylation	11	22.1	4.67E-09	2.19E-08
R-HSA-389957:R-HSA-389957	Protein folding	Prefoldin mediated transfer of substrate to CCT/TriC	11	19.0	2.91E-08	1.37E-07
R-HSA-389960:R-HSA-389960	Protein folding	Formation of tubulin folding intermediates by CCT/TriC	10	17.9	5.30E-07	2.49E-06
R-HSA-156902:R-HSA-156902	Protein synthesis	Peptide chain elongation	32	17.0	8.00E-28	3.76E-27
R-HSA-192823:R-HSA-192823	Disease	Viral mRNA Translation	31	16.4	2.29E-26	1.07E-25
R-HSA-2408557:R-HSA-2408557	Metabolism of amino acids	Selenocysteine synthesis	32	16.4	2.56E-27	1.20E-26
R-HSA-72764:R-HSA-72764	Protein synthesis	Eukaryotic Translation Termination	31	15.8	1.00E-25	4.70E-25
R-HSA-975956:R-HSA-975956	Protein synthesis	Nonsense Mediated Decay (NMD) independent of the Exon Juncti	31	15.4	2.04E-25	9.56E-25
R-HSA-70171:R-HSA-70171	Glucose metabolism	Glycolysis	10	15.1	2.90E-06	1.36E-05
R-HSA-70263:R-HSA-70263	Glucose metabolism	Gluconeogenesis	10	15.1	2.90E-06	1.36E-05
R-HSA-72689:R-HSA-72689	Protein synthesis	Formation of a pool of free 40S subunits	32	14.9	9.04E-26	4.24E-25
R-HSA-5625740:R-HSA-5625740	Signal transduction	RHO GTPases activate PKNs	8	13.8	3.26E-04	0.00153
R-HSA-156827:R-HSA-156827	Protein synthesis	L13a-mediated translational silencing of Ceruloplasmin expressior	32	13.5	2.12E-24	9.96E-24
R-HSA-72706:R-HSA-72706	Protein synthesis	GTP hydrolysis and joining of the 60S ribosomal subunit	32	13.4	2.85E-24	1.34E-23
R-HSA-1799339:R-HSA-1799339	Protein synthesis	SRP-dependent cotranslational protein targeting to membrane	31	13.1	4.39E-23	2.06E-22
R-HSA-975957:R-HSA-975957	Protein synthesis	Nonsense Mediated Decay (NMD) enhanced by the Exon Junctior	31	13.0	5.84E-23	2.74E-22
R-HSA-72695:R-HSA-72695	Protein synthesis	Formation of the ternary complex, and subsequently, the 43S com	14	12.5	1.19E-08	5.58E-08
R-HSA-72702:R-HSA-72702	Protein synthesis	Ribosomal scanning and start codon recognition	14	11.1	6.21E-08	2.91E-07
R-HSA-72649:R-HSA-72649	Protein synthesis	Translation initiation complex formation	14	11.1	6.21E-08	2.91E-07
R-HSA-445355:R-HSA-445355	Musce contraction	Smooth Muscle Contraction	8	11.0	0.0016393	0.0077
R-HSA-5663213:R-HSA-5663213	Signal transduction	RHO GTPases Activate WASPs and WAVEs	8	10.7	0.0019995	0.00939
R-HSA-3928662:R-HSA-3928662	Signal transduction	EPHB-mediated forward signaling	9	10.3	5.11E-04	0.0024
R-HSA-450408:R-HSA-450408	Metabolism of RNA	AUF1 (hnRNP D0) binds and destabilizes mRNA	11	9.5	4.79E-05	2.25E-04
R-HSA-5358346:R-HSA-5358346	Signal transduction	Hedgehog ligand biogenesis	11	8.8	9.44E-05	4.43E-04
R-HSA-211733:R-HSA-211733	Programmed cell death	Regulation of activated PAK-2p34 by proteasome mediated degra	9	8.7	0.0020186	0.00948
R-HSA-1236978:R-HSA-1236978	Immune system	Cross-presentation of soluble exogenous antigens (endosomes)	9	8.7	0.0020186	0.00948
R-HSA-5362768:R-HSA-5362768	Disease	Hh mutants that don't undergo autocatalytic processing are degra	10	8.6	5.12E-04	0.0024
R-HSA-69481:R-HSA-69481	Cell cycle	G2/M Checkpoints	9	8.5	0.0023519	0.01105
R-HSA-350562:R-HSA-350562	Metabolims of polyamines	Regulation of ornithine decarboxylase (ODC)	9	8.5	0.0023519	0.01105
R-HSA-69229:R-HSA-69229	Cell cycle	Ubiquitin-dependent degradation of Cyclin D1	9	8.5	0.0023519	0.01105
R-HSA-69017:R-HSA-69017	Cell cycle	CDK-mediated phosphorylation and removal of Cdc6	9	8.5	0.0023519	0.01105
R-HSA-180534:R-HSA-180534	Disease	Vpu mediated degradation of CD4	9	8.4	0.0027306	0.01283
R-HSA-349425:R-HSA-349425	Cell cycle	Autodegradation of the E3 ubiquitin ligase COP1	9	8.4	0.0027306	0.01283
R-HSA-69601:R-HSA-69601	Cell cycle	Ubiquitin Mediated Degradation of Phosphorylated Cdc25A	9	8.2	0.0031592	0.01485
R-HSA-180585:R-HSA-180585	Disease	Vif-mediated degradation of APOBEC3G	9	8.0	0.0036431	0.01713
R-HSA-5678895:R-HSA-5678895	Disease	Defective CFTR causes cystic fibrosis	10	7.9	0.001072	0.00503
R-HSA-4641257:R-HSA-4641257	Proteolysis	Degradation of AXIN	9	7.9	0.0041878	0.0197

R-HSA-174113:R-HSA-174113	Proteolysis	SCF-beta-TrCP mediated degradation of Emi1	9	7.9	0.0041878	0.0197
R-HSA-4641258:R-HSA-4641258	Proteolysis	Degradation of DVL	9	7.6	0.0054834	0.02581
R-HSA-68827:R-HSA-68827	DNA replication	CDT1 association with the CDC6:ORC:origin complex	9	7.4	0.0070986	0.03343
R-HSA-5676590:R-HSA-5676590	Signal transduction	NIK-->noncanonical NF-kB signaling	9	7.4	0.0070986	0.03343
R-HSA-5610783:R-HSA-5610783	Proteolysis	Degradation of GLI2 by the proteasome	9	7.2	0.0080442	0.0379
R-HSA-187577:R-HSA-187577	Proteolysis	SCF(Skp2)-mediated degradation of p27/p21	9	7.2	0.0080442	0.0379
R-HSA-5610780:R-HSA-5610780	Proteolysis	Degradation of GLI1 by the proteasome	9	7.2	0.0080442	0.0379
R-HSA-5607761:R-HSA-5607761	Signal transduction	Dectin-1 mediated noncanonical NF-kB signaling	9	7.2	0.0080442	0.0379
R-HSA-5610785:R-HSA-5610785	Proteolysis	GLI3 is processed to GLI3R by the proteasome	9	7.2	0.0080442	0.0379
R-HSA-1236974:R-HSA-1236974	Immune system	ER-Phagosome pathway	11	6.3	0.0022507	0.01058

Table S5 Real Time PCR primers

Gene	Forward	Reverse
HK1	CTGCTGGTGAAAATCCGTAGTGG	GTCCAAGAAGTCAGAGATGCAGG
HK2	TCCACTCCTCTCAGCATTGG	CTTGCCCCATTATCCCATGC
GLO1	ATGCGACCCAGAGTTACCAC	CCAGGCCTTTCATTTTACCA
ACTB	GGACTTCGAGCAAGAGATGG	AGCACTGTGTTGGCGTACA

Gel immunoblotting originals.

Fig. 2h (Glo1 protein and β-actin reference) and Fig. 2m (Glo1 protein band used for normalisation of ubiquitinated Glo1).

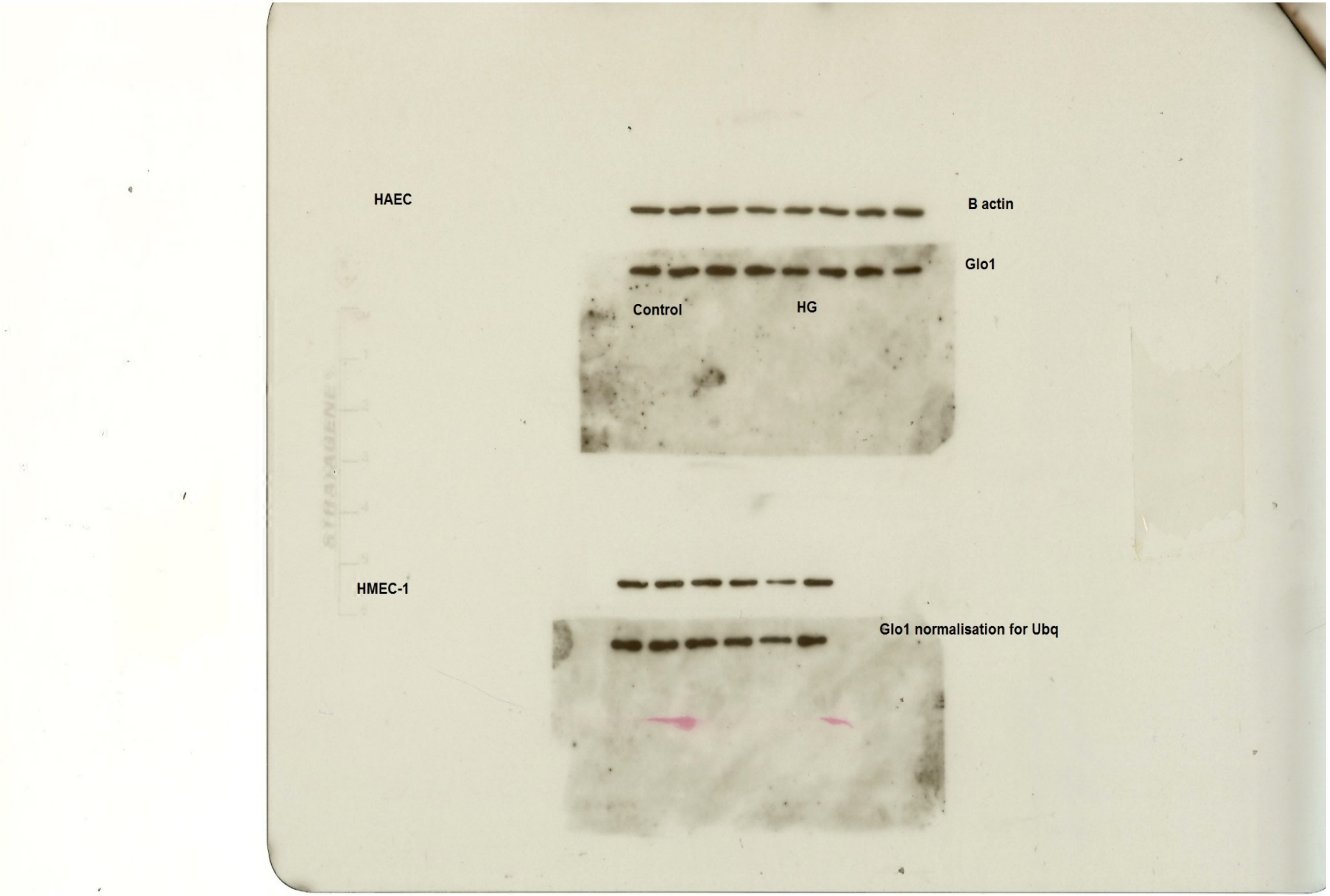

Fig. 2k (Glo1 protein and β-actin reference)

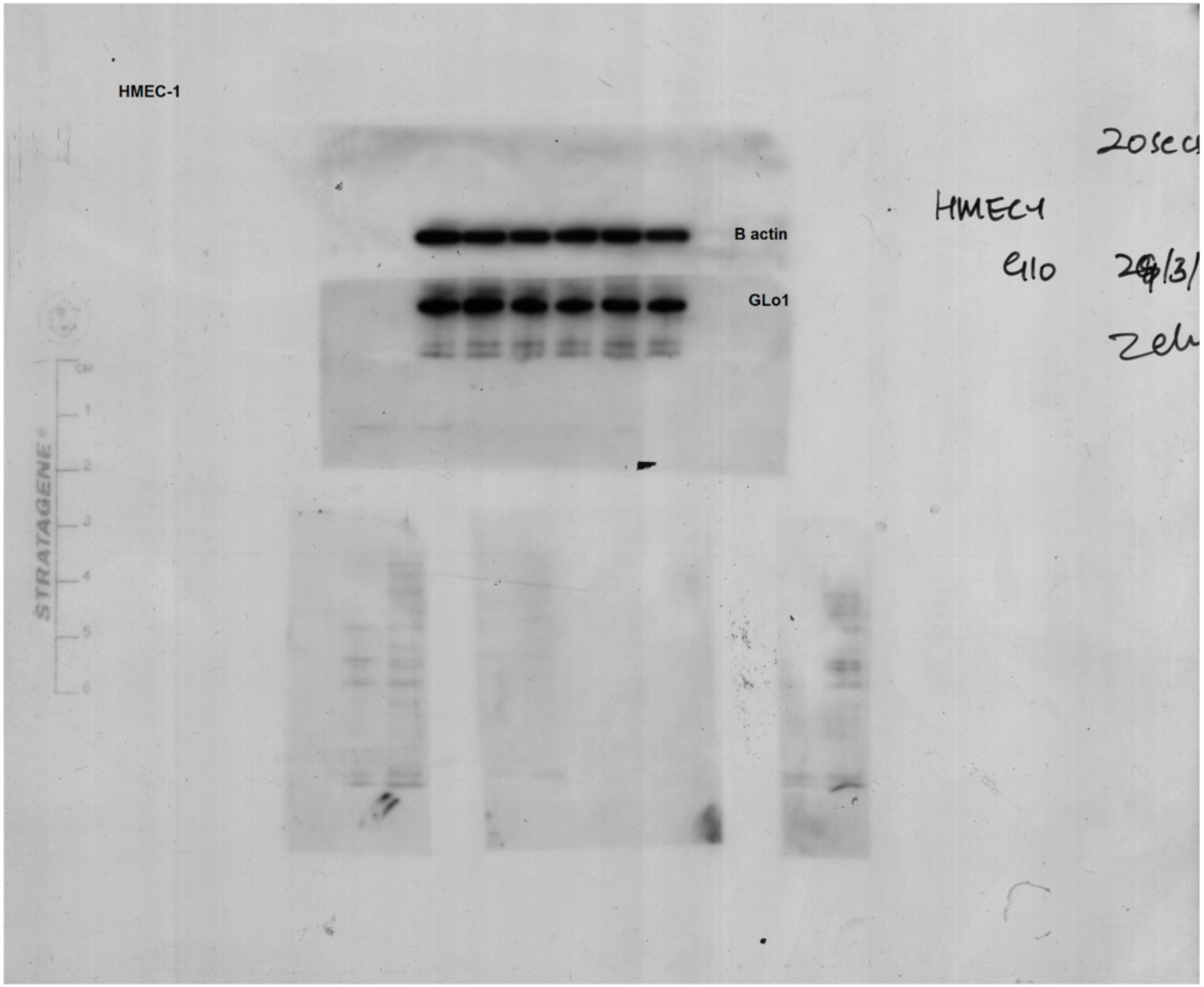

Fig. 2m (Ubiquitinated Glo1 protein band)

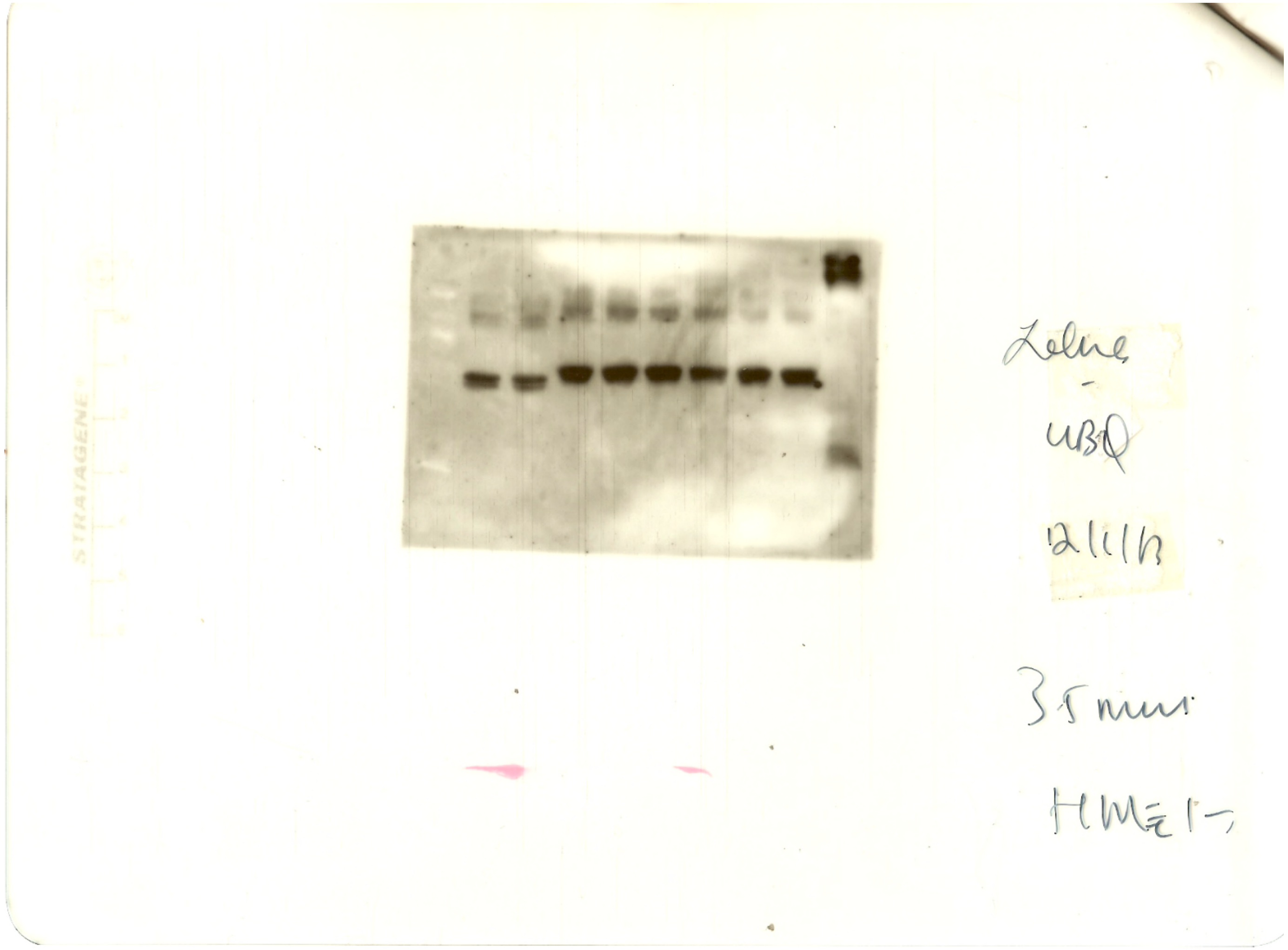

Fig. 3a. HSP70 – 3 days and 6 days, low and high glucose

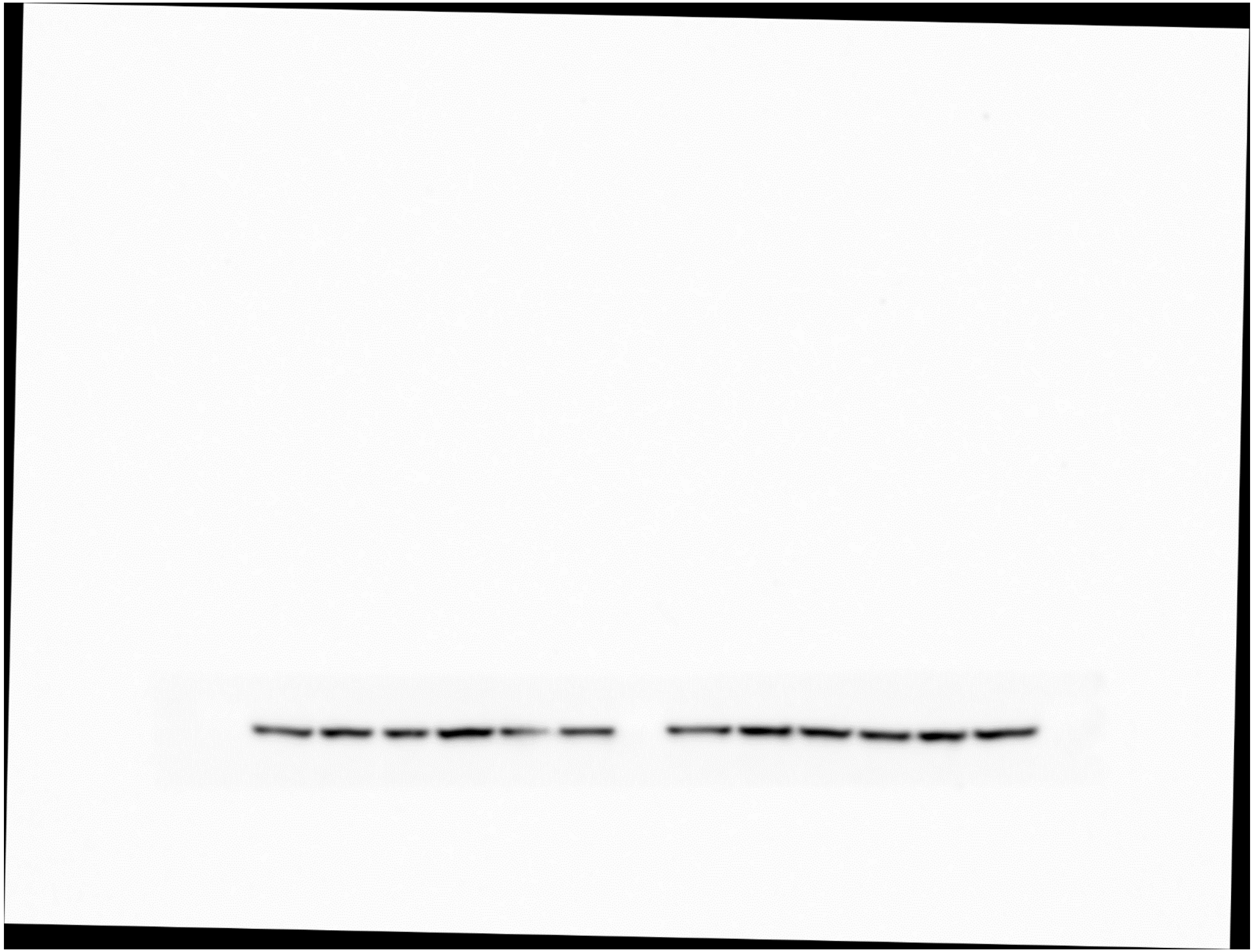

Fig. 3a. GRP78 – 3 days and 6 days, low and high glucose

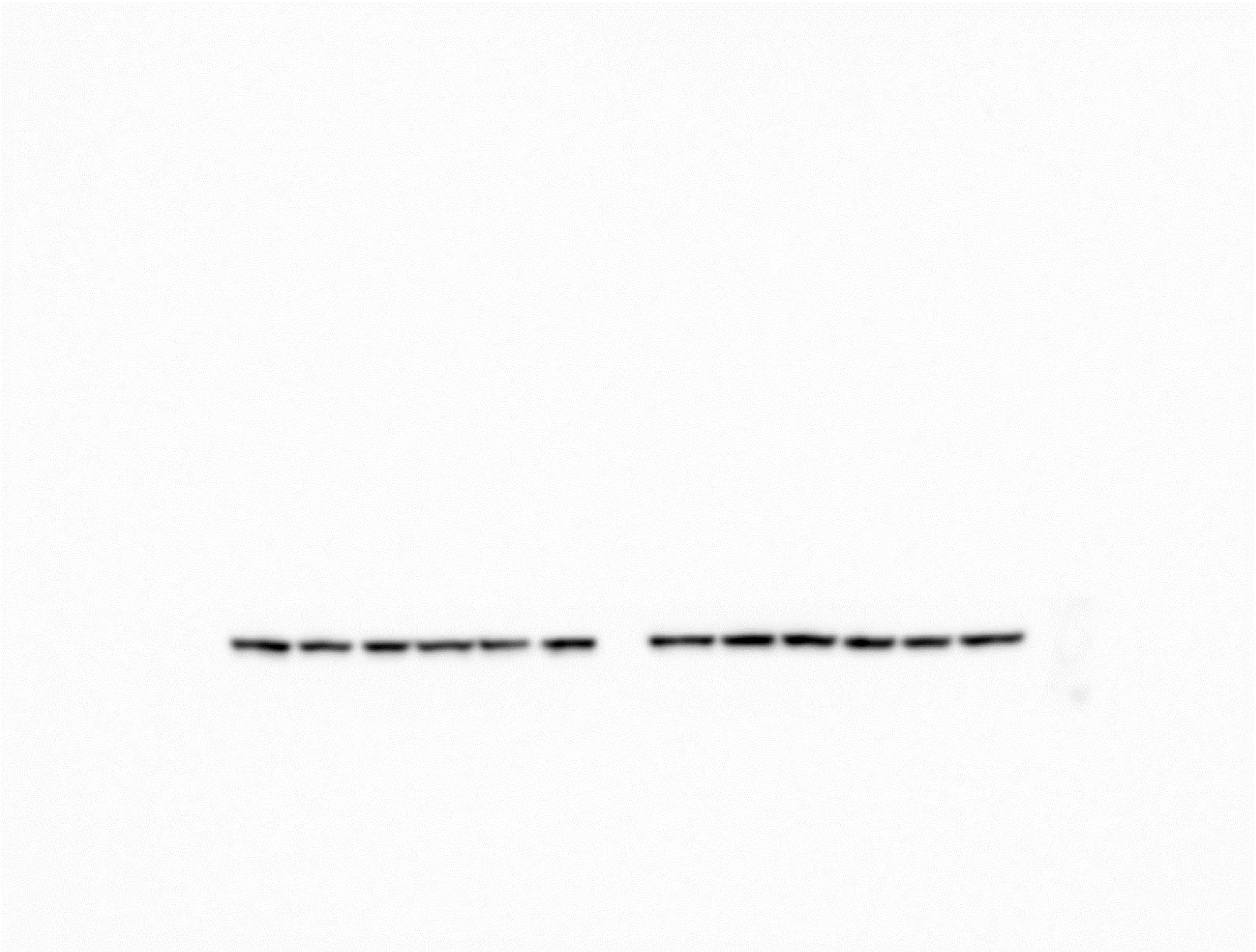

Fig. 3a. β-Actin reference band – 3 days and 6 days, low and high glucose

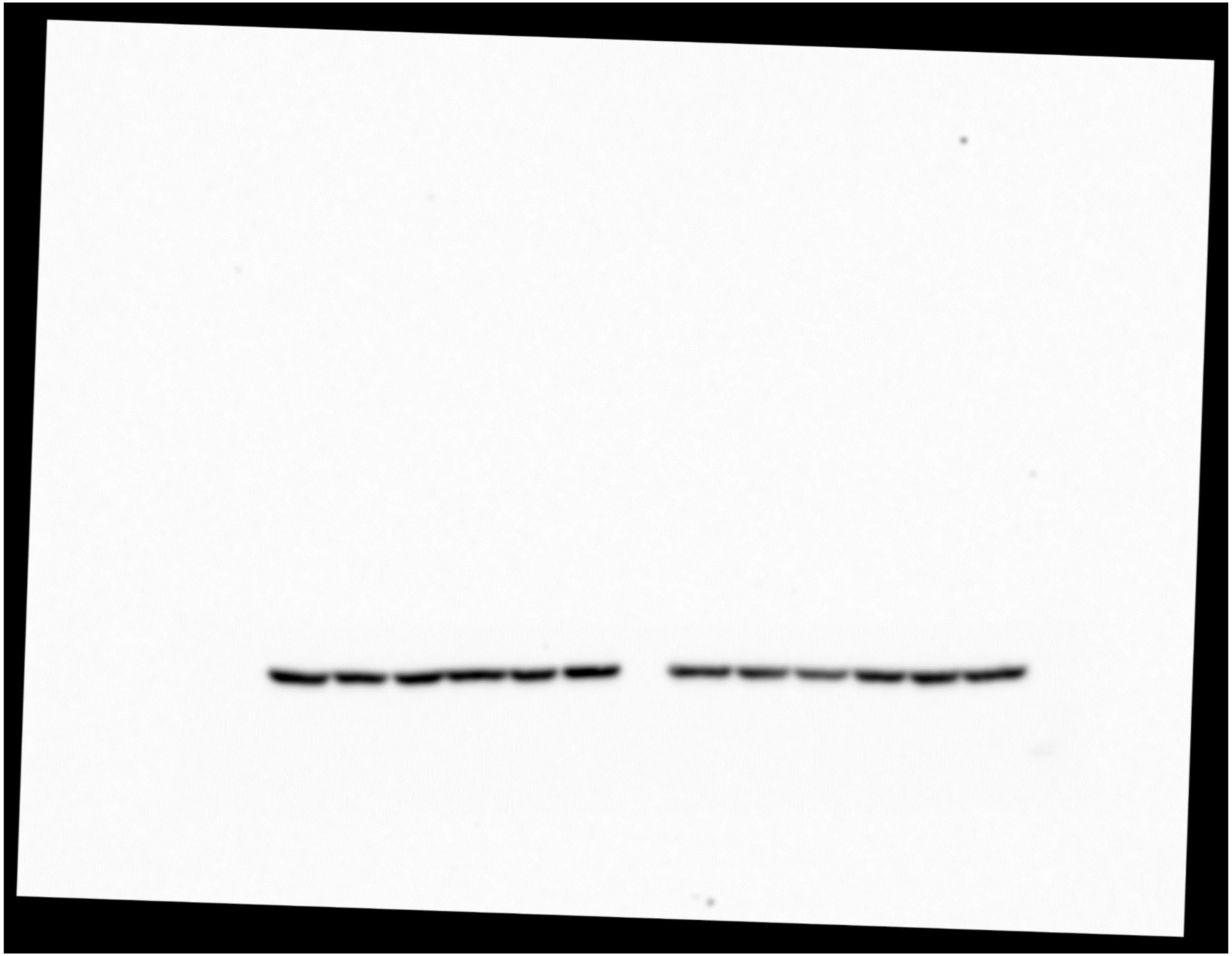

Fig. 3d. Immunoblotting for Glo1. Top gel is used in the manuscript.

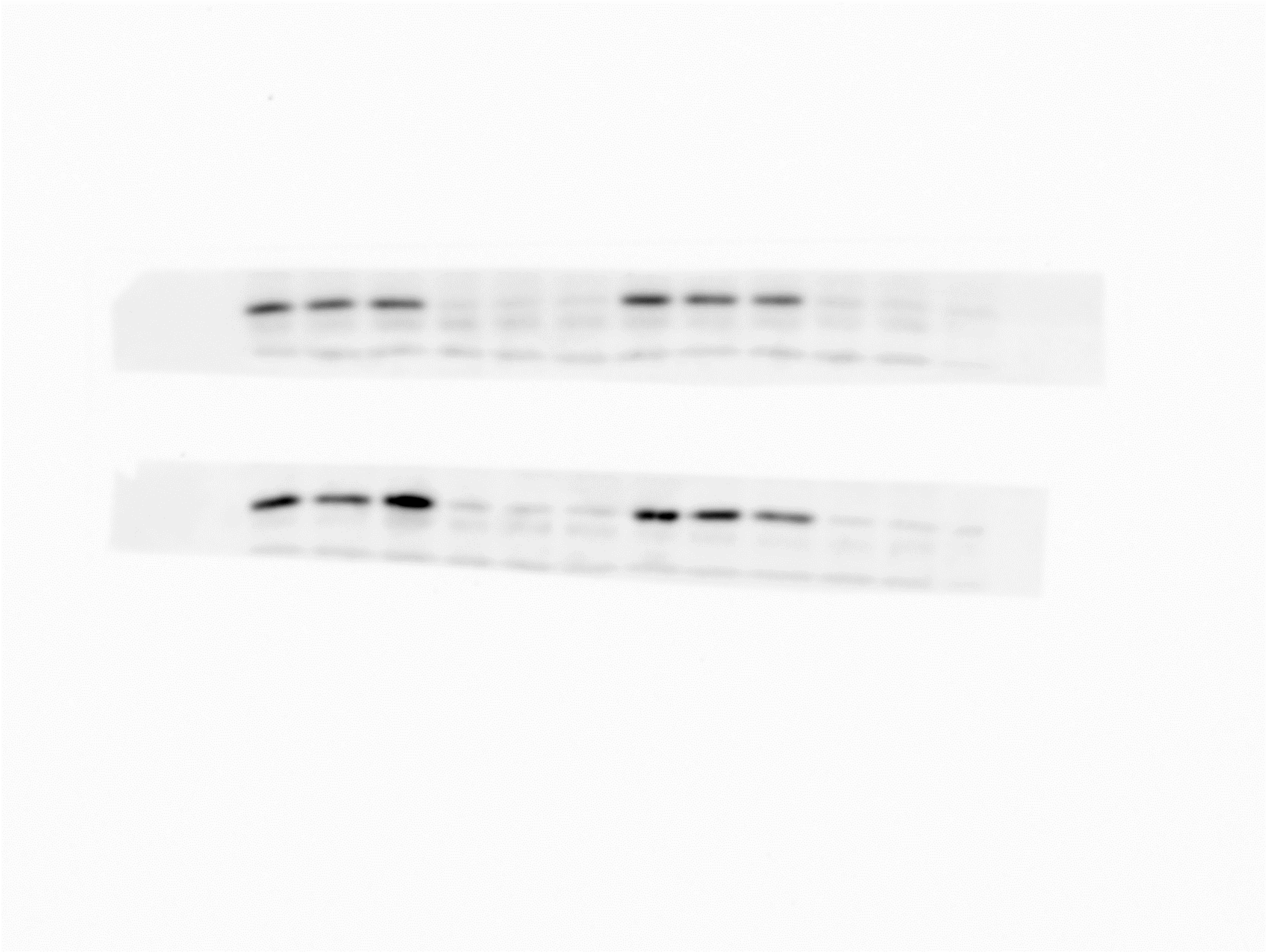

Fig. 3d. Immunoblotting for HSP70.

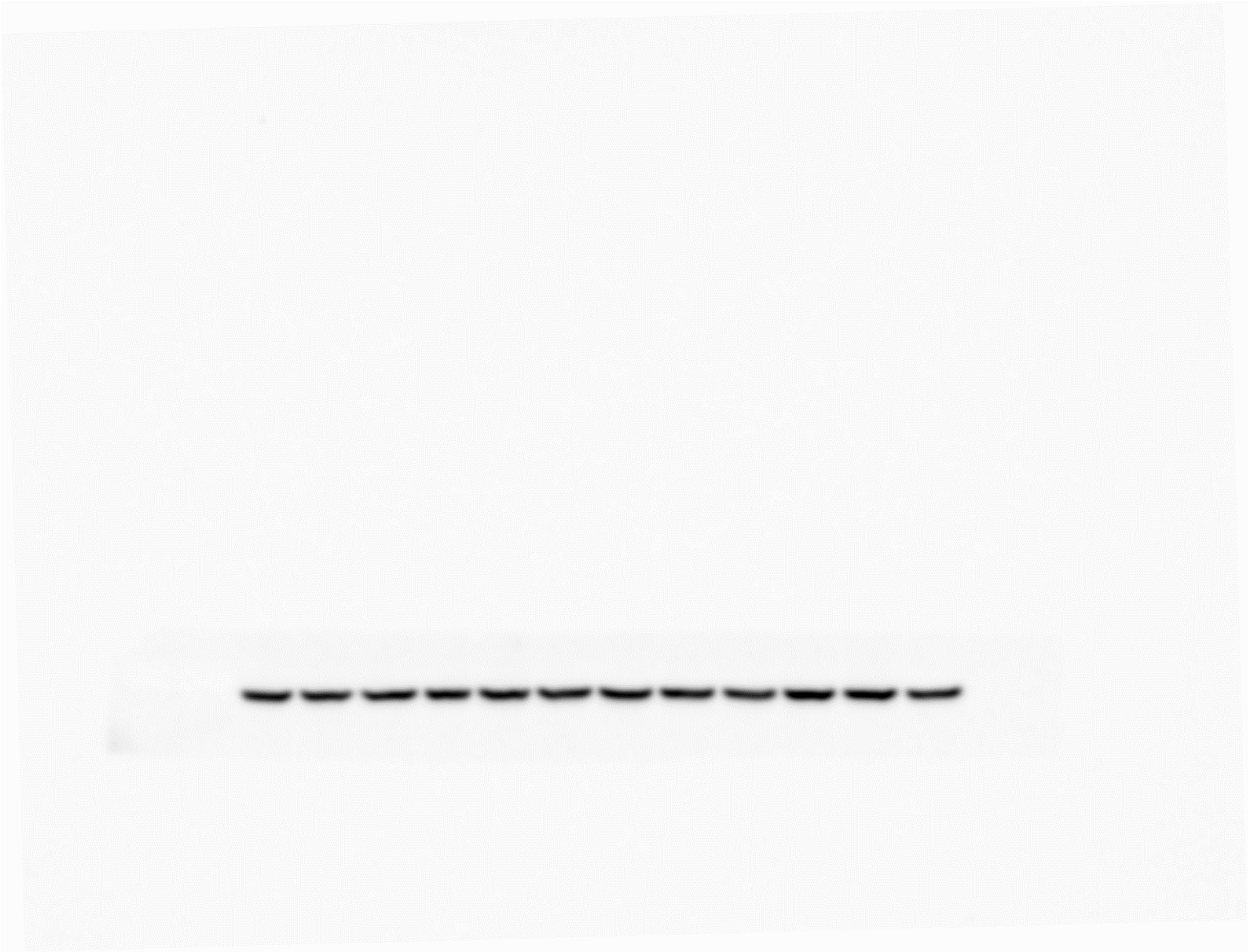

Fig. 3d. Immunoblotting for GRP78.

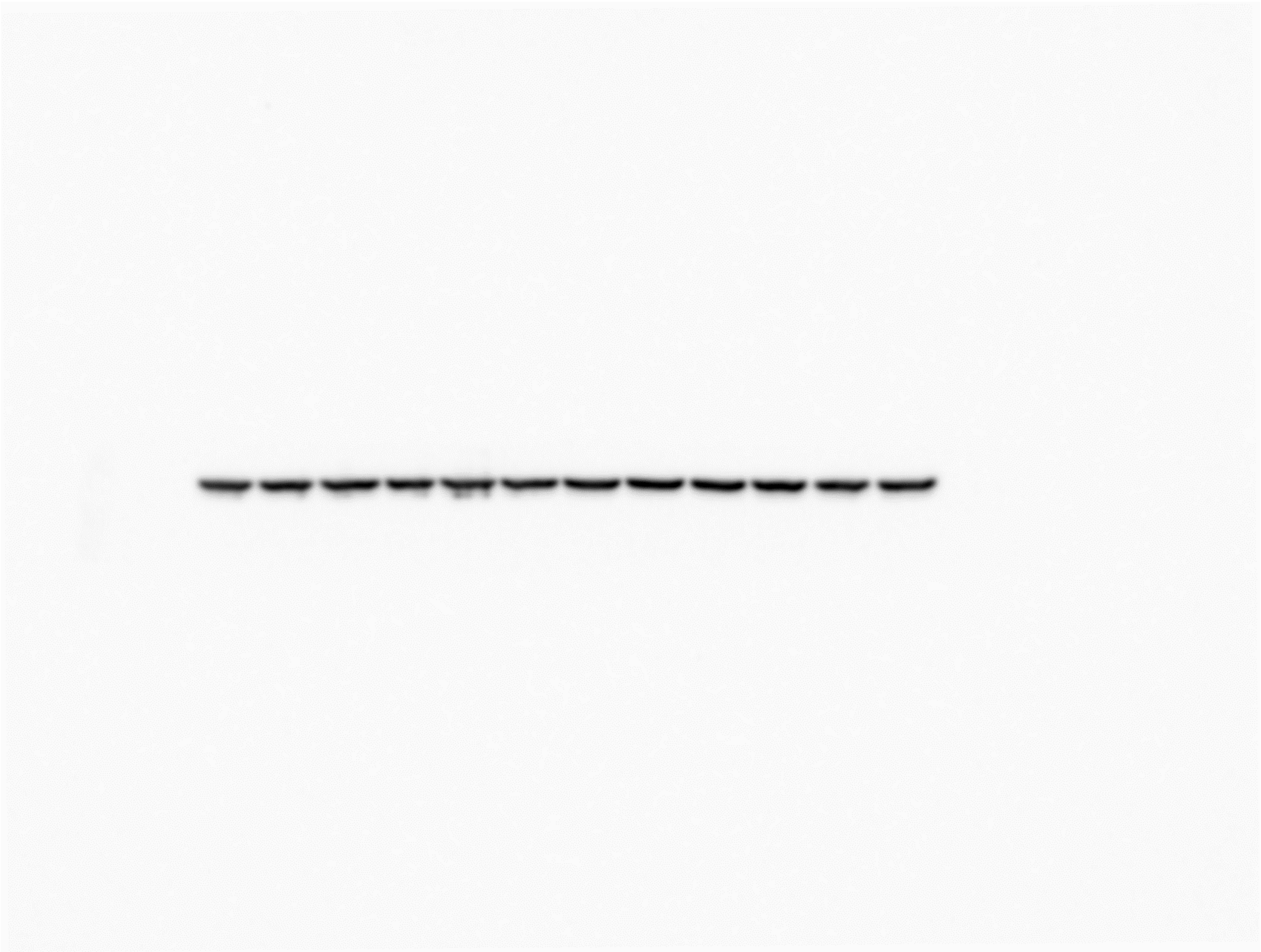

Fig. 3d. Immunoblotting for β-actin reference.

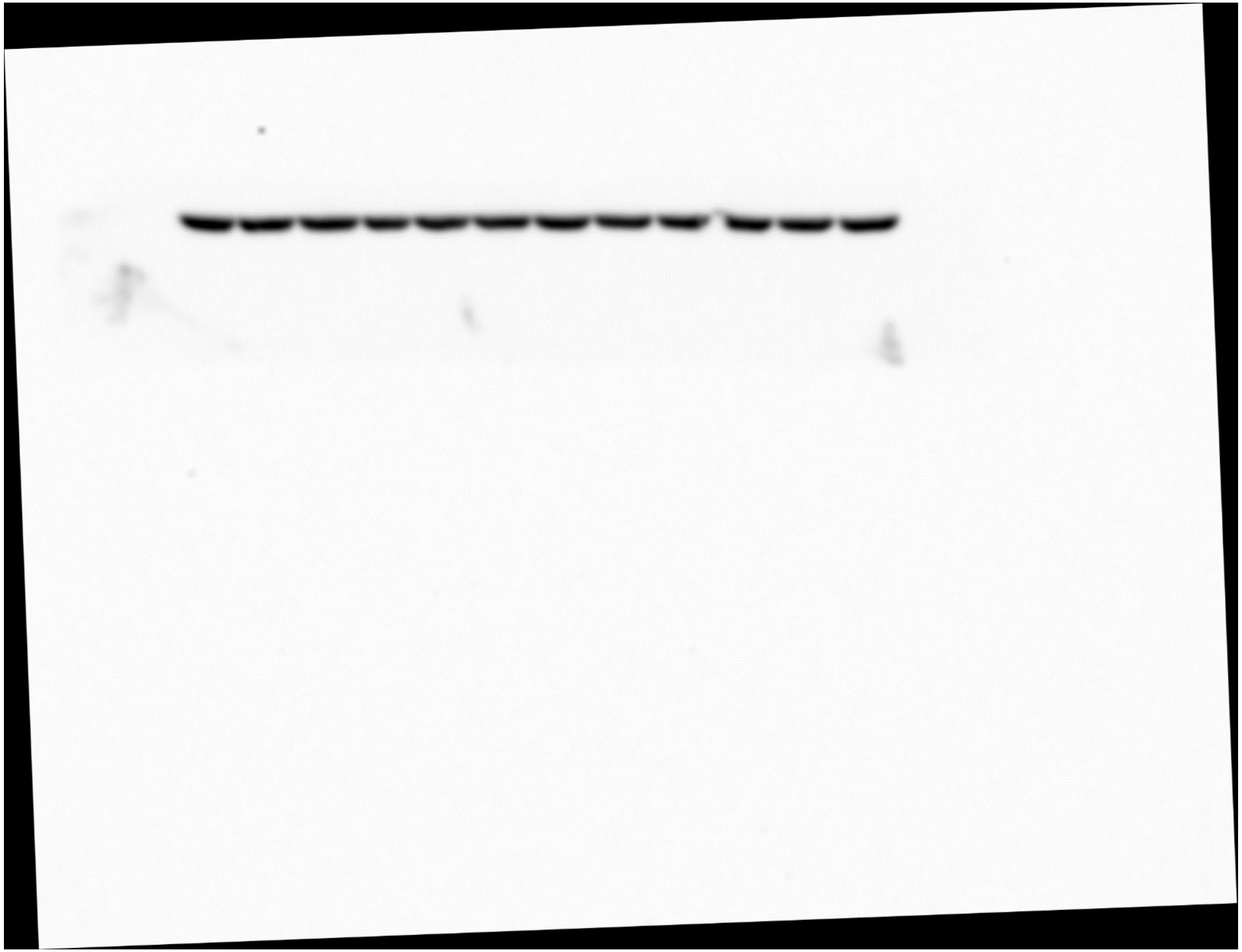

Fig. 5b. HK-2 – 3 days and 6 days, low and high glucose [bottom membrane & band]. (β-Actin reference band is given above – same as for Fig. 3a)

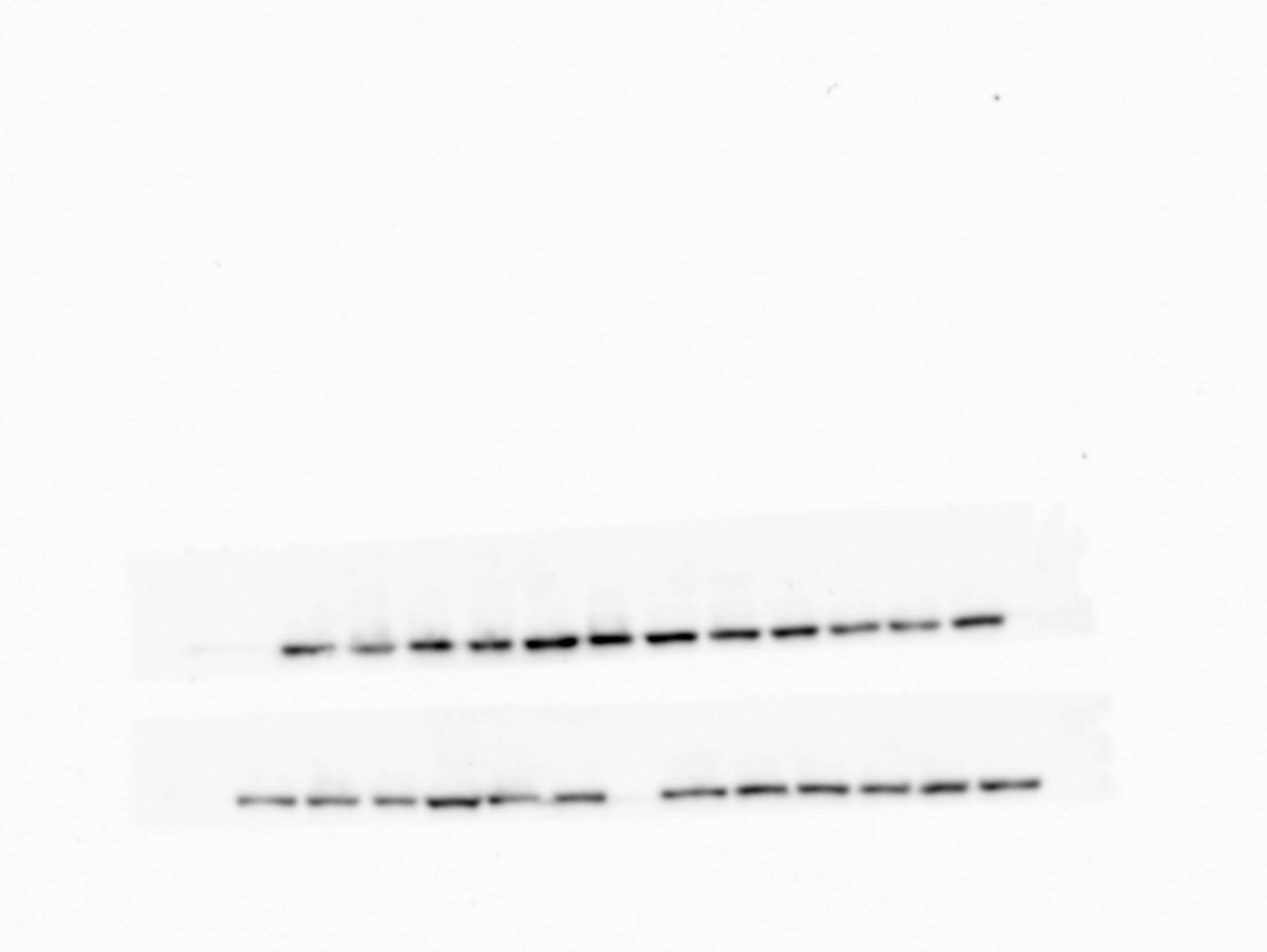

Fig. 5f. HK-2 knockdown study. HK-2 antibody immunoblotting (top) and β-Actin reference (bottom).

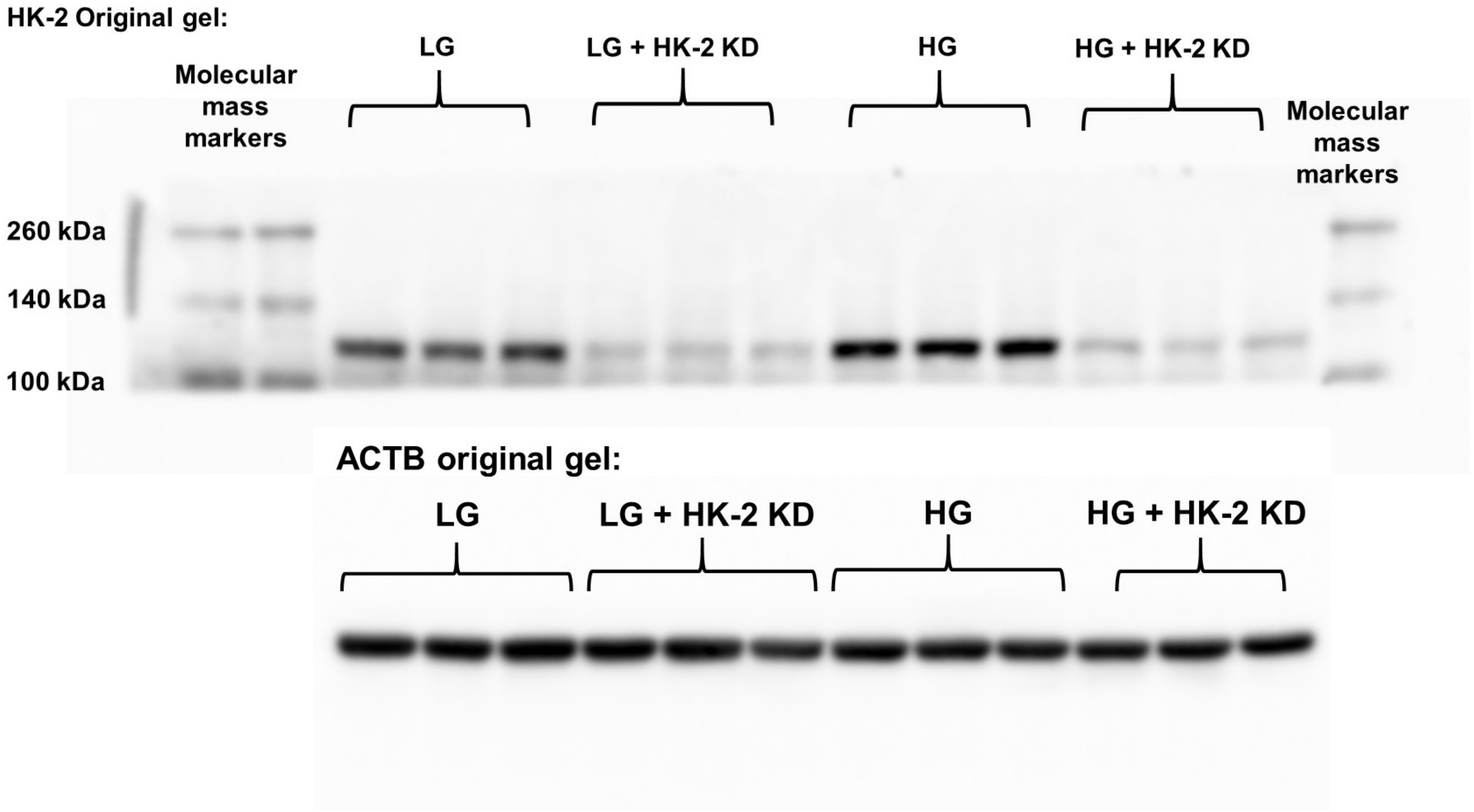

Fig. 6e. Immunoblotting for G6PD.

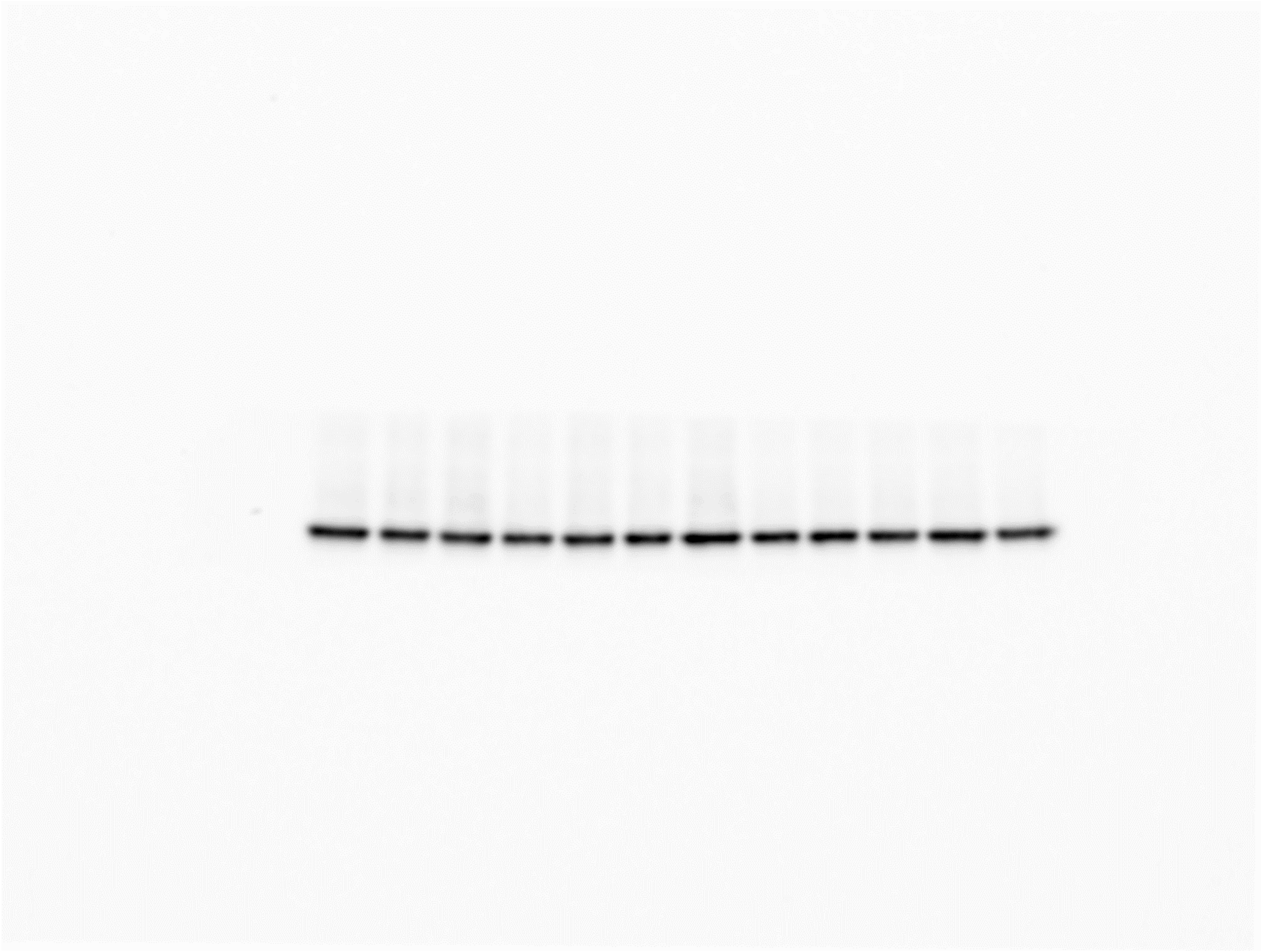

Fig. 6e. Immunoblotting for G6PD: β-actin reference.

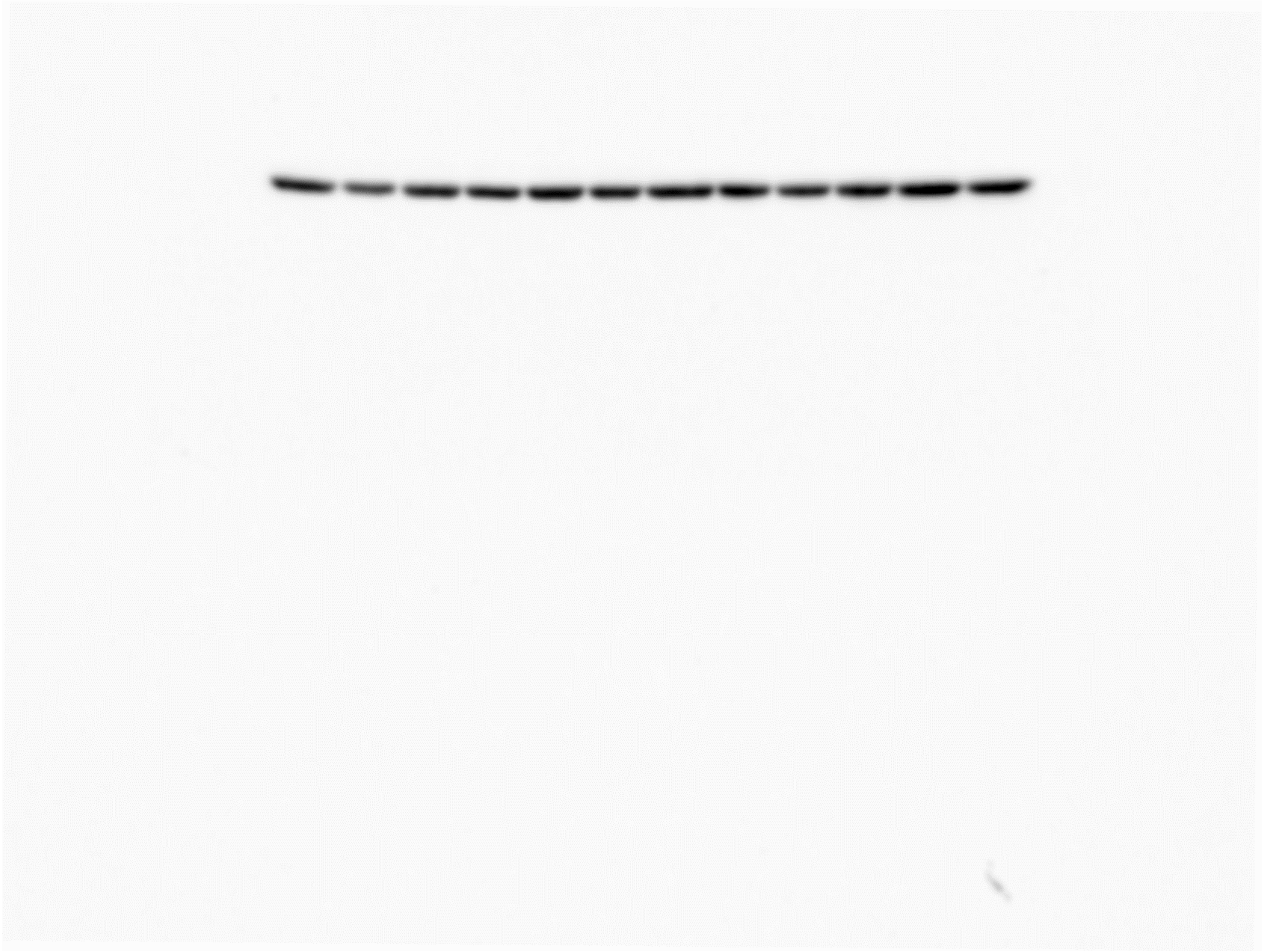

Fig. 6f. Immunoblotting for HK-2.

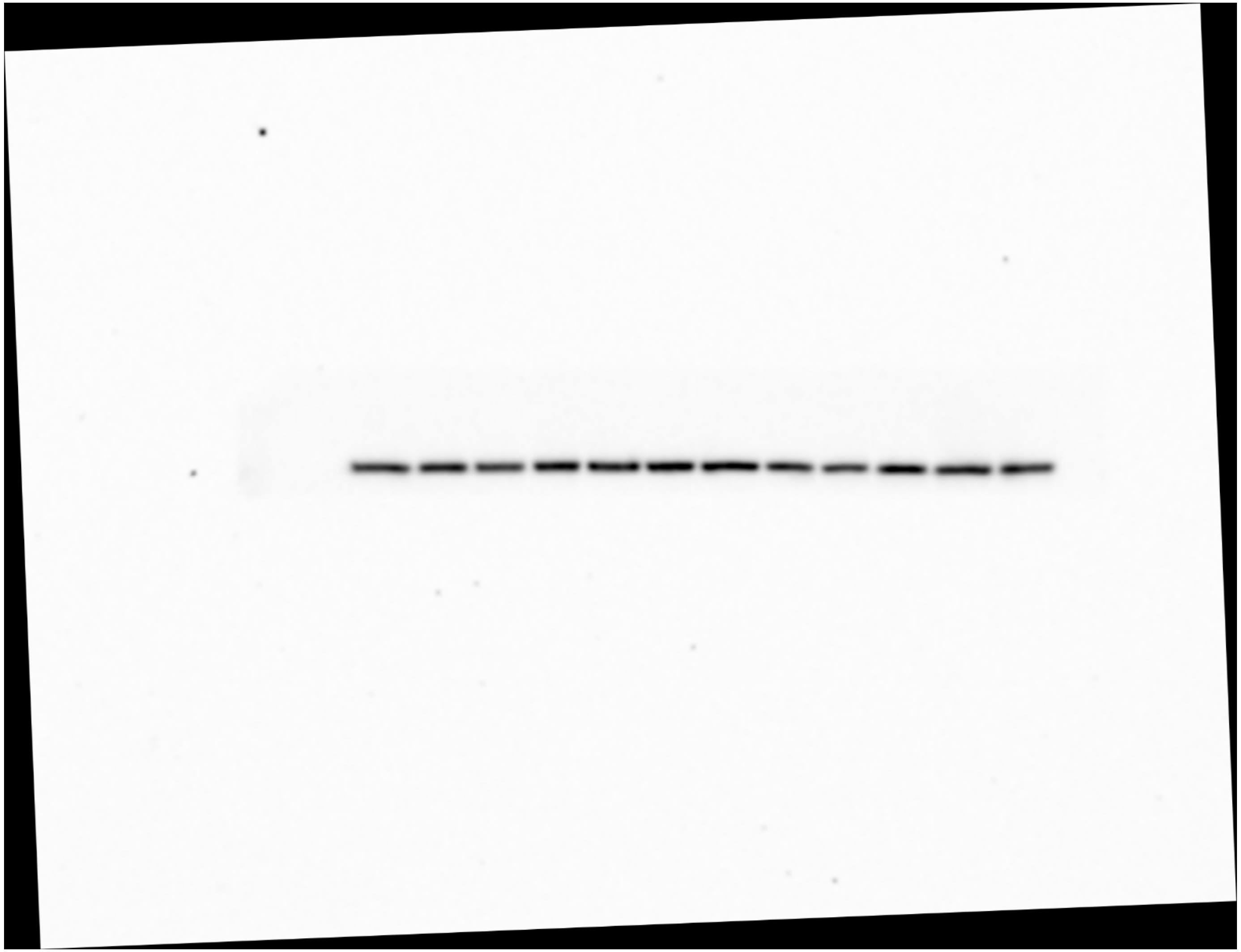

Fig. 6f. Immunoblotting for HK-2: β-actin reference.

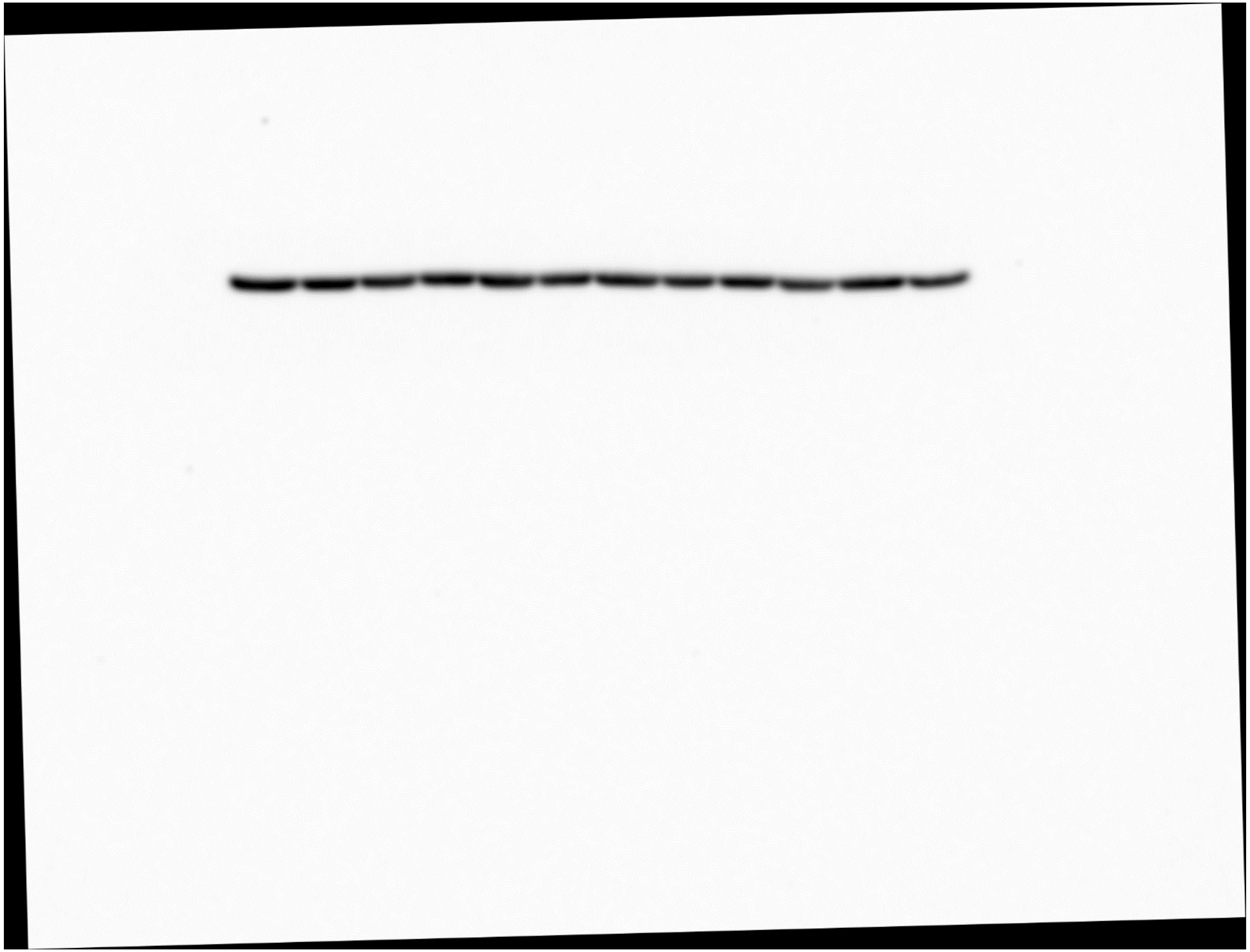

Supplement: Supplementary file 1 — Supplementary information, tables and original gel images [file 41598_2019_44358_MOESM1_ESM.pdf]
